# “Finally my turn to write my story”: A convergent mixed methods study exploring the perceptions and experiences of emerging adults who aged out of foster care in Canada

**DOI:** 10.1371/journal.pone.0338574

**Published:** 2025-12-29

**Authors:** Brianna Jackson, Margaret L. Holland, Victoria Smye, Sarah Lowe, Lois S. Sadler

**Affiliations:** 1 School of Nursing, Yale University, New Haven, Connecticut, United States of America; 2 School of Nursing, University of Wisconsin-Madison, Madison, Wisconsin, United States of America; 3 School of Health Sciences, University of New Haven, New Haven, Connecticut, United States of America; 4 Arthur Labatt Family School of Nursing, Western University, London, Ontario, Canada; 5 School of Public Health, Yale University, New Haven, Connecticut, United States of America; Khyber Medical University, PAKISTAN

## Abstract

**Background:**

Each year, thousands of young Canadians ‘age out’ of foster care on or before their 19^th^ birthday. This abrupt transition to independence coincides with emerging adulthood (ages 18–29), a developmental period associated with transformational changes, including new or worsening mental health challenges.

**Objective:**

The purpose of this study was to understand how emerging adults, who aged out of foster care in Canada, navigated their transition to independence, and specifically, how experiences of structural violence encountered pre- and post-emancipation may have influenced their mental health and capacity for positive adaptation.

**Participants and Setting:**

203 emerging adults from across Canada took part in the quantitative arm of this study, with a subsample of 31 participants enrolled in the qualitative arm. Virtual methods (online survey and video conferencing) supported remote participation.

**Methods:**

A convergent mixed methods design involved concurrent quantitative and qualitative data collection and analysis, followed by integration of emergent findings. The quantitative arm of the study consisted of an electronic questionnaire including sociodemographic characteristics, foster care histories, and ten validated measures. The qualitative arm involved virtual semi-structured interviews regarding participants’ transition to independence upon aging out of care, including experiences and perceptions of structural violence, mental health challenges, and positive adaptation.

**Results:**

Correlation analyses and regression modelling revealed relationships between and among structural violence, mental health challenges, and positive adaptation in this population. Nine qualitative themes uncovered the contextual nuances of participants’ transition to independence. Two joint displays were developed to visually represent the integration of quantitative and qualitative data.

**Conclusions:**

By exploring this clinical issue from a combined socioecological, temporal, and intersectional perspective, key findings reflect its complexity, nuance, and transformative capacity. Integrated data may suggest approaches for future development of interventions to address the unique mental health care needs of this population.

## Background

### Introduction

Of the estimated 78,000 youth in the Canadian foster care system [1], approximately half will become permanent wards—characterized by long-term placement in government custody—and will ‘age out’ of care on or before their 19th birthday [2]. Forced to grow up seemingly overnight, those who are emancipated from foster care are stripped of the structured supports to which they have become accustomed [3,4], and are left to navigate complex social services with minimal guidance and financial resources [5,6].

For many, this abrupt transition to independence is associated with a sharp and steady functional decline [7], as evidenced by limited educational attainment, unemployment, housing instability, tumultuous relationships, early parenthood, criminal involvement, substance use, as well as poor physical and mental health [8–20]. Coinciding with the developmental period of emerging adulthood—most commonly defined as spanning ages 18–29 [21,22]—many youth aging out of foster care experience higher-than-average rates of depressive, anxiety, and post-traumatic stress symptomatology than their peers [23–26].

Emerging adulthood is a pivotal stage of growth, maturation, and exploration, evidenced by substantial physical, relational, and sociopolitical development [22,27]. Associated with the reduction or loss of various supports, services, and resources [28], as well as an expanded range of social freedoms, this period is also marked by greater self-reliance and independent decision-making [21]. As a period of significant neurobiological development that coincides with changing social roles and responsibilities, emerging adulthood often aligns with the onset of new or worsening mental health challenges [23,24,26,29,30].

### Significance

Researchers have found that biobehavioral development and life course trajectories are influenced by both intrinsic (e.g., genetics, personality traits) and extrinsic factors (e.g., social relationships, physical environment) [31–34]; however, few studies have explored the cumulative biopsychosocial effects of chronic adversity faced by emerging adults who have aged out of foster care. These effects include deeply rooted systemic challenges and barriers that threaten longevity, health-related quality of life, and access to opportunity [35–38].

This persistent and toxic form of stress, termed structural violence, arises when populations marginalized by inequity are disproportionately harmed by institutional policies and practices [39,40], owing to an inequitable distribution of wealth, power, and resources [41]. Interdisciplinary experts agree upon the deleterious sequelae of structural violence, both broadly [42,43] and with respect to mental health and wellbeing [32–34]; however, positive adaptive responses to prolonged adversity within this population have been minimally examined [44]. A more comprehensive and nuanced understanding of positive adaptation, including its risk and protective factors, may not only help to identify key barriers and facilitators to overcoming adversity, but may also reveal opportunities for proactive and preventive interventions that address broader sources of structural violence.

Given a lack of nationally representative descriptive data pertaining to Canada’s foster children, thorough investigation is urgently needed in order to understand the clinical needs of those who have aged out of care. Existing supportive and mental health services are seldom tailored to this specific population, and often lack consideration of contextual complexities, such as the layered effects of multiple marginalizing identities, combined with experiences of childhood and ongoing trauma and violence [45]. Exploring the intersection of emerging adulthood, structural violence, mental health, and positive adaptation may advance clinical knowledge and supportive policies by uncovering and describing the multifaceted nature of this threat to health and wellbeing.

### Conceptual underpinnings

Situated within an emancipatory paradigm [46], this exploratory study is both equity-informed and justice-oriented, drawing upon principles of critical social theory [47,48] to interrogate the structural forces that shape health and wellbeing. The research is anchored in three complementary frameworks: Bronfenbrenner’s [49] socioecological model, Crenshaw’s [50] intersectionality framework, and Halfon and Hochstein’s [51] life course health development model—which together provide systemic, intersectional, and temporal lenses for examining how structural violence influences the mental health and positive adaptation of emerging adults aging out of foster care.

**Fig 1:**
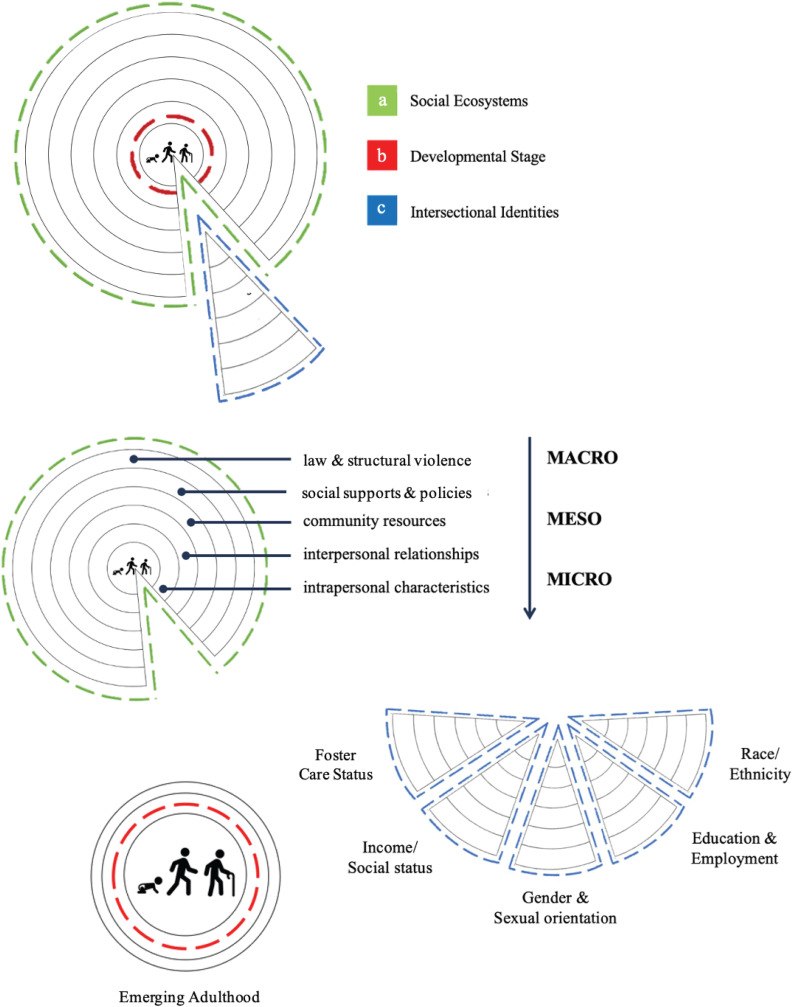
Conceptual model.

Bronfenbrenner’s model situates individual outcomes within nested and bidirectional ecological systems that shape and are shaped by human development. Crenshaw’s intersectionality framework highlights how multiple, interlocking identities—such as race, gender, sexuality, and social status—compound exposure to marginalization and adversity. The life course health development model emphasizes how biobehavioral trajectories evolve through dynamic interactions with social, physical, and natural environments over time and across generations. Integrating these perspectives supports a mixed methods study capable of linking multilevel determinants to lived experience; thereby generating nuanced, contextually grounded insights into this complex clinical phenomenon (Fig 1).

### Purpose

The purpose of this study was to understand how emerging adults (18–29) who aged out of foster care in Canada, navigated their transition to independence. By exploring how experiences of structural violence influenced mental health and wellbeing, and in turn identifying intrinsic and extrinsic factors that both promoted and impeded positive adaptation, lessons learned from former youth-in-care may illuminate opportunities for the future development and implementation of mental health care that is tailored to the unique clinical needs of this population.

## Methods

### Design

**Fig 2:**
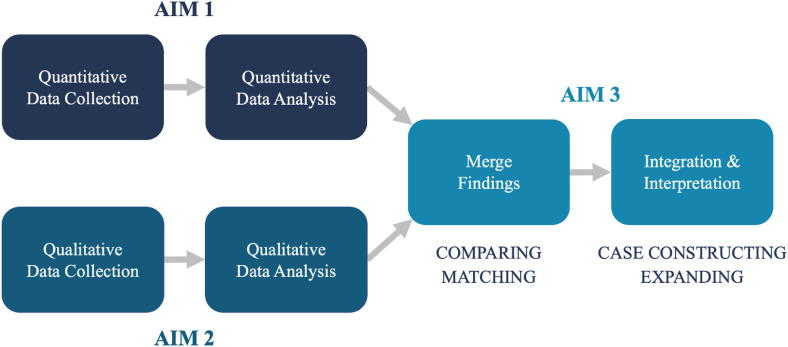
Mixed Methods Study Design.

To address the stated purpose, a convergent mixed methods design was used [52,53], in which quantitative and qualitative data collection and analysis occurred concurrently (Fig 2). Data were then merged and reviewed, with the final point of integration involving the joint interpretation, display, and dissemination of mixed methods findings. Thorne’s [54] interpretive description approach was used to guide qualitative investigation and analysis, given its constructivist and naturalistic orientation, which prioritizes the generation of clinically relevant knowledge [55]. Interpretive description seamlessly integrates empirics and aesthetics; thereby supporting a comprehensive understanding of complex phenomena and associated processes, suitable for uptake by health professions.

Three aims included quantitative, qualitative, and mixed (integrated) methods, respectively. For a sample of emerging adults (18–29) who aged out of foster care in Canada, the study aims were to:

1. Describe and explore associations among: (a) experiences of structural violence (adverse childhood experiences, intersectional discrimination, perceived stress); (b) protective factors (benevolent childhood experiences); (c) mental health challenges (post-traumatic stress, anxiety, depression); and (d) positive adaptation (resilience, flourishing). (Quantitative)
2. Explore descriptions and overall perceptions of: (a) the transition to independence; (b) experiences of structural violence; (c) relationships among adverse life experiences, protective factors, and positive adaptation; and (d) clinical mental health needs, including suggestions for improved care. (Qualitative)
3. Generate a comprehensive understanding of relationships between and among: (a) experiences of structural violence; (b) protective factors; (c) mental health challenges; and (d) positive adaptation, through the integration of quantitative and qualitative data. (Mixed Methods)

For the quantitative arm of the study, participants were surveyed with an electronic questionnaire including sociodemographic characteristics, foster care history, and ten validated measures (Table 1). The qualitative arm involved virtual semi-structured interviews regarding participants’ transition to independence upon aging out of care, including experiences and perceptions of structural violence, mental health challenges, and positive adaptation. Data collection extended from February 17 to August 31, 2023.

**Table 1:** Variables and Measures.

CONCEPT	VARIABLE	MEASURE
***Structural Violence***	*Childhood Adversity*	Adverse Childhood Experiences (ACEs) Questionnaire [10]
	*Discrimination*	Intersectional Discrimination Index (InDI): Anticipated (InDI-A) [9] and Day-to-Day (InDI-D) [9]
	*Perceived Stress*	Perceived Stress Scale (PSS) [10]
***Mental Health***	*Post-Traumatic Stress*	Abbreviated PTSD Checklist for DSM-5 (PCL8−5) [8]
	*Anxiety*	PROMIS Anxiety Short Form 4a [4]
	*Depression*	PROMIS Depression Short Form 6a [6]
***Positive Adaptation***	*Resilience*	Brief Resilience Scale (BRS) [6]
	*Flourishing*	Secure Flourishing Index (SFI) [12]
***Protective Factors***	*Positive Childhood Experiences*	Benevolent Childhood Experiences (BCEs) Questionnaire [10]

*Note:* Number of items per instrument indicated in parentheses. Combined total of 84 items across all measures.

### Participants and setting

A total of 203 emerging adults (ages 18–29) who aged out of foster care in Canada participated in the quantitative arm of this study, with a subsample of 31 participants in the qualitative arm. All study procedures took place remotely, and recruitment efforts targeted emerging adults across all Canadian provinces and territories.

#### Sampling strategy.

A purposive sampling approach that incorporated snowball and opportunistic strategies was used to recruit participants. An electronic flyer was shared with community agencies, support services, and advocacy organizations that served Canadian former youth-in-care specifically, or indirectly, as part of another marginalized group (e.g., unhoused youth). The same flyer was also featured in targeted social media posts on Facebook and Instagram. All advertisements included a QR code and link to a brief summary of the study, as well as the email address and business telephone number of the lead author.

Potential participants were required to meet the following eligibility criteria for inclusion in the study:

1. Able to read, write, and converse fluently in English
2. Between the ages of 18 and 29 at the time of enrollment
3. Former permanent ward placed in foster care in any Canadian province or territory
4. Aged out of foster care in Canada upon reaching the legal age of child protection (16–19), as defined by the respective jurisdiction
5. Remained a resident of Canada from the time of emancipation until study commencement

Those interested in the interview portion of the study were required to meet two additional criteria:

1. Access to an electronic device with a video camera and internet connectivity, such as a smart phone, tablet, or personal computer
2. Agreed to the audio-recording and transcription of interview dialogue

While 203 participants were ultimately enrolled in the study, a modest sample size of 50 was initially targeted. Given the exploratory nature of the study design, effect size was prioritized over statistical significance. Using confidence intervals (CI) and effect size to guide sampling, power analyses conducted using a medium effect of 0.5 and a liberal 80% CI, consistently confirmed the suitability of a sample size ≥50 for exploratory purposes. However, anticipated logistical challenges related to the recruitment, retention, and engagement of the target population were not encountered, allowing for a much larger sample than originally proposed. To support future study comparisons, the original power calculation was adjusted to reflect a 95% CI, which demonstrated the need for a sample size of approximately 64 participants. This remains far below the actual sample size (*n* = 203) achieved for this study.

At the time of enrollment, participants were invited to take part in both the quantitative and qualitative arms of this study; however, involvement in the interview process was dependent upon participants’ decision to ‘opt in’ following survey completion. An information power approach was used to determine the final size of the interview subsample [56]. Owing to high participant specificity and strong interview dialogue, among other factors, sufficient information power was achieved earlier than anticipated. Consistent with established methodological precedent among studies that use an interpretive description approach [54], 31 interviews were conducted, of which 26 transcripts were cleaned and analyzed. Included participants were selected purposively to maximize the representation of diverse intersectional identities and lived experiences.

### Ethical considerations

Ethics approval for this study was obtained from the Human Subjects Committee of Yale University’s Institutional Review Board (IRB # 2000033580). Since the study was grounded within a Canadian context, all procedures maintained alignment with the Government of Canada’s ‘Tri-Council Policy on Ethical Conduct for Research Involving Humans’, with particular attention to guidelines for ‘Research Involving the First Nations, Inuit, and Métis Peoples of Canada’ [57], as well as the First Nations principles of Ownership, Control, Access, and Possession (OCAP) [58]. Consistent with these principles, the research team collaborated with organizations that serve Indigenous communities in order to facilitate recruitment, ensure voluntary participation, and uphold cultural safety throughout all stages of the research process. All potential participants were sent an electronic copy of the study’s Letter of Information (LOI) and were given the opportunity to express any concerns and ask questions before providing their consent to participate through the online survey.

### Quantitative arm

#### Quantitative data collection.

Participants completed a self-administered online questionnaire through Qualtrics XM [59]. Surveys included multiple choice and Likert-style questions about participants’ personal characteristics and foster care histories, as well as ten validated self-report measures assessing experiences of structural violence (Adverse Childhood Experiences, Intersectional Discrimination Index–Anticipated and Day-to-Day, Perceived Stress Scale), protective factors (Benevolent Childhood Experiences), mental health challenges (Abbreviated PTSD Checklist for DSM-5, PROMIS Emotional Distress and Anxiety Short Form 4a, and PROMIS Depression Short Form 6a), and positive adaptation (Brief Resilience Scale and Secure Flourishing Index) (Table 1). The study surveys were pilot-tested with an individual who was representative of the study sample. Surveys took approximately 15 minutes to complete and could be paused and resumed at any time. Participants received a $30 CAD electronic gift card following submission of the questionnaire.

##### Sociodemographic and Foster Care Characteristics.

Participants were asked about their age, race, Indigeneity, first language, gender identity, sexual orientation, relationship status, parental status, social supports, educational attainment, employment status, financial resources, living arrangements, and disability status. The online survey also contained information about their time in foster care, including: the reason they entered the child welfare system, duration of time in foster care, total number of placements, province/territory of residency, age upon system entry and exit, period of eligibility for supports post-emancipation, and types of transitional supports provided.

##### Variables and Measures.

**Structural Violence:** The Adverse Childhood Experiences (ACEs) questionnaire [60,61] is comprised of 10 items that reflect the presence of different childhood adversities experienced from ages 0–18, including abuse and neglect, household dysfunction and instability, and exposure to domestic violence. Cronbach’s alpha of 0.88 indicates reliable and valid internal consistency of the ACEs questionnaire [62].

The Intersectional Discrimination Index–Anticipated (InDI-A) is a 9-item questionnaire that measures respondents’ current beliefs about how their combined personal identities might influence inequitable and unfair treatment across relational and structural domains. The InDI-A has high internal consistency (*α* = 0.93), and test-retest reliability (*r* = 0.72) exceeds that of comparable discrimination measures [63].

The Intersectional Discrimination Index–Day-to-Day (InDI-D) measures lifetime discrimination experienced on a daily basis, with nine items assessing the frequency of unwarranted negative behaviors by others. Test-retest reliability for the InDI-D (r = 0.70) is similar to that of the InDI-A [63].

The Perceived Stress Scale (PSS) is a 10-item questionnaire that assesses difficulty managing and coping with current stress [64]. In a study of college students, Cronbach’s alpha was 0.89, indicating high internal consistency reliability [65]. Test-retest reliability ranges from moderate to high (r = 0.74–0.88) across studies [66].

**Protective Factors:** The Benevolent Childhood Experiences (BCEs) questionnaire is a 10-item retrospective self-report measure of positive experiences occurring from ages 0–18. Items assess perceived relational and internal safety and security, positive and predictive quality of life, and interpersonal support [67]. This measure has shown high internal consistency (*a* = 0.80) [68], test-retest reliability (*r* = 0.91) [69], and good predictive validity for psychopathology and stressful life events across diverse adult samples [70].

**Mental Health Challenges:** The 8-item abbreviated post-traumatic stress disorder (PTSD) checklist for the fifth edition of the Diagnostic and Statistical Manual of Mental Disorders (DSM-5)—referred to as the PCL8−5—is a self-report instrument that measures the degree to which participants have been bothered by symptoms of PTSD during the past month. Each of the eight items is drawn from the original 20-item version of the PCL-5 [71]. The condensed PCL8−5 measure has demonstrated comparable psychometric properties to the longer PCL-5 [72], with highly correlated scores, as well as strong internal consistency (α = 0.93) and test-retest reliability (*r* = 0.84) [73].

The PROMIS Emotional Distress-Anxiety Short Form 4a is a brief 4-item self-report measure that broadly assesses fear, worry, hyperarousal, and somatizations associated with anxiety [74]. With each item corresponding to an anxiety-related feeling or experience, respondents are prompted to report the frequency of such symptoms within the past seven days. Studies involving the PROMIS Emotional Distress-Anxiety Short Form 4a [75] dependably report moderate to high internal consistency reliability (a = 0.79–0.89) [76] and strong correlations with other standardized anxiety measures.

The PROMIS Depression Short Form 6a is a brief self-report measure featuring six Likert-style questions that collectively assess negative alterations in mood, affect, self-perception, engagement, and social cognition [77]. With each item reflecting a particular depressive symptom, respondents are asked to indicate how often they experienced such symptoms within the past seven days using a 5-point rating scale. Reliability estimates are consistently high across diverse samples (*a* = > 0.90) [78]. The measure demonstrates excellent internal consistency (*a* = 0.95) and shows a very strong correlation with the PROMIS Depression Full 28-Item Bank (r = 0.95–0.96) [79,80].

**Positive Adaptation:** The Brief Resilience Scale (BRS) is a 6-item self-report measure assessing the degree to which participants are able to return to their previous level of functioning following a stressful experience. Among samples of young adults, the BRS has demonstrated good internal consistency (a = 0.84–0.87) [81].

The Secure Flourishing Index (SFI) is a 12-item self-report measure designed to assess six domains of human flourishing: happiness and life satisfaction; mental and physical health; meaning and purpose; character and virtue; close social relationships; and financial and material stability [82]. Psychometric evaluation by Węziak-Białowolska and colleagues [83,84] has demonstrated strong support for both convergent and discriminant validity across all six domains, with high test-retest correlations (*r* = 0.73–0.87) indicating stability over time. Further study has confirmed the internal consistency and reliability (*α* = 0.86) of the SFI [85].

#### Quantitative data analysis.

All quantitative data were cleaned and imported into SAS 9.4 [86] for analysis. Descriptive statistics were calculated to describe sample characteristics and variables of interest, assess the normality of data distribution, identify possible outliers, and any missing data. With a total Fraction of Missing Information [87] of only 3.6%, it was determined that a multiple imputation approach was not needed to treat missing data [88]. A correlation matrix was developed to examine bivariate associations. Those with moderate to strong correlation coefficients (*r* ≥ 0.3) were included in subsequent regression analyses (Table 5).

**Fig 3:**
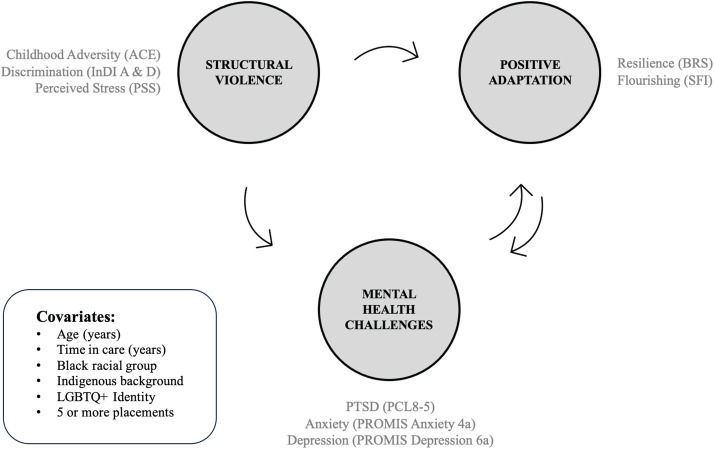
Variable Associations Tested Through Regression Modelling.

Multiple linear regression analyses were conducted with a block sequential approach for the direction and strength of relationships among quantitative variables, when controlling for six covariates (age, total time in care, Black racial background, Indigenous ethnicity, LGBTQ+ status, and a history of five or more foster care placements). These covariates were selected since they were unique intersectional identities that may have meaningfully influenced participants’ lived experiences and perceptions [50]. Ten different associations were examined through regression modelling (Fig 3).

### Qualitative Arm

#### Qualitative data collection.

Thirty-one participants completed individual semi-structured interviews guided by an interpretive description approach [54]. A preliminary interview guide was developed, pilot-tested, and revised with help from an individual who met study eligibility criteria. Refinement of the interview guide was an iterative process, and prompts were continually adjusted based upon quantitative findings and ongoing qualitative interview experiences.

All interviews were conducted remotely using Zoom.us [89] video conferencing software and were approximately 45–60 minutes in length. Interviews were conducted by the first author—a PhD Candidate and Psychiatric-Mental Health Registered Nurse with qualitative research and clinical interviewing experience. Prior to the interview, introductions and explanations of the study were reviewed and informed consent was obtained. For a complete description of virtual methods and participant safeguards, see Jackson et al. (manuscript under review). Field notes were made following each interview. Participants received a second $30 CAD electronic gift card for engaging in the interview process. Participants were reimbursed for any travel, childcare, or phone charges incurred, up to a maximum of $15 CAD. Half of the interview subsample (*n* = 13) was invited to review emergent theme descriptions as part of member-checking, which took place after thematic analysis was complete. Participants were purposively selected to reflect diverse sociodemographic characteristics and foster care experiences.

#### Qualitative data analysis.

Interview dialogue was audio-recorded through Zoom.us [89] and a back-up digital recording device. It was then transcribed verbatim, using Temi [90] advanced speech recognition software. Transcripts were reviewed for accuracy, cleaned, and de-identified. Inductive thematic analysis of interview dialogue was used to describe how the conceptual model (Fig 1)—comprised of three converging theoretical frameworks—manifested in this unique clinical context [91]. Following Thorne’s [54] interpretive description framework, qualitative analysis proceeded through five iterative phases: (1) immersion in data and open coding; (2) identification and categorization of patterns; (3) development of interpretive themes through constant comparison; (4) synthesis and integration of thematic relationships; and, (5) validation through team consensus and participant member-checking. The cross-sectional and emergent nature of this study supported concurrent analysis that coincided with ongoing recruitment and continued revision of the semi-structured interview guide, based upon participant and researcher experiences during preceding interviews. This process yielded rich thematic findings that informed integration with quantitative results.

All qualitative analysis activities were conducted by the first author and senior (last) author. Blinded transcripts were open coded, the coding structure was discussed for consensus, and all transcripts were imported into ATLAS.ti software, version 24.1.0 [92] for further analysis. An iterative codebook contained detailed definitions of codes and subcodes. Following review of the first five transcripts, initial codes were compared to establish an integrated axial coding structure and to assess intercoder agreement. A consensus of 80% was not achieved, so all remaining interview transcripts were co-coded by both researchers. Codes were clustered into networks and overarching categories. This process continued until no new information could be gathered, and sufficient richness of data was achieved.

Themes were developed by combining categories, and were defined using a thick description technique. Exemplar quotations were selected to represent each theme. Following thorough examination, revision, reflection, and explication [91], resulting theme descriptions were shared with thirteen interested participants for their review and comment. Two participants responded and agreed with the themes presented, and they were highly enthusiastic about the qualitative findings.

### Mixed methods integration

Integration took place at two distinct points during the study (Fig 2), and involved strategies of *comparing, matching, case constructing,* and *expanding* [52,53,93]. Following concurrent analysis, preliminary quantitative and qualitative findings were merged for *comparing* in order to identify convergent and divergent findings. This stage also involved *matching* at the individual level by pairing participants’ subjective experiences with corresponding sociodemographic characteristics, foster care histories, and instrument scores.

At the second point of integration, all data were jointly interpreted by examining how findings related to initial research questions, overarching theoretical frameworks, the devised conceptual model, extant literature, and a personal assessment of meaning. Following a comprehensive review of the merged results, *case constructing* was used to assemble participant profiles and identify divergent case exemplars representing diverse participant characteristics and experiences.

Connections between quantitative constructs and qualitative themes were drawn to visually represent relationships among different types of data. Key quantitative and qualitative findings were then featured in two joint displays (Figs 5 and 6), which encompassed quantitative variables and descriptive statistics, qualitative themes and illustrative quotations, as well as meta-inferences pulled from key integrated findings [52]. This comprehensive integration strategy provided a nuanced and holistic representation of results, conclusions, and implications [94].

**Fig 4:**
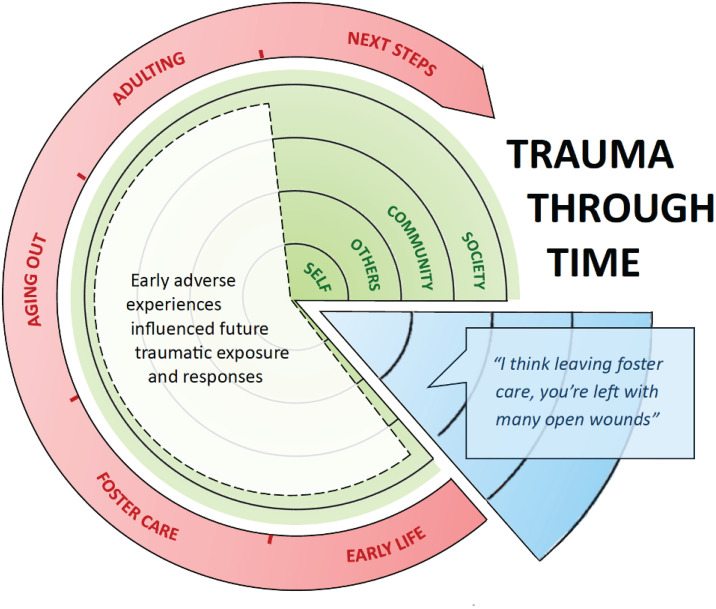
Qualitative Theme Exemplar: Trauma Through Time.

### Rigor and trustworthiness

Various strategies were used to enhance methodological rigor during each phase of the study. For the quantitative arm, validated measures and instruments with strong internal consistency were included. Lincoln and Guba’s [55] four trustworthiness criteria—*credibility, transferability, dependability,* and *confirmability—*and the consolidated criteria for reporting qualitative research (COREQ) were used to increase transparency [95]. *Credibility* was supported by the triangulation of data sources, collection strategies, and theoretical positionality. An audit trail encompassing field notes, a detailed codebook, memos, and correspondence with participants and community partners was developed; thereby maintaining referential adequacy. Member-checking practices helped to validate qualitative findings and contextualized description of themes supported the *transferability* of study findings. Research team members’ co-coding and the study’s emergent design strengthened *dependability*. Guidance by the senior author, as well as self-reflexivity—encompassing the acknowledgement of personal biases, and efforts to maintain neutrality—addressed *confirmability*. For the mixed methods phase, four different integration techniques (*comparing, matching, case constructing,* and *expanding*) were used at various points throughout the study, and the theoretical underpinnings and study conceptual framework formed the lens through which the research problem was understood, the purpose of the investigation was derived, and the design was constructed.

## Results

### Participant characteristics

The survey sample (*n* = 203) was quite diverse, with respect to sociodemographics (Table 2) and foster care experience (Table 3). While many respondents (33.9%) self-identified as White, all seven racial groups were reflected, with people of color collectively representing the majority of the sample (76.6%). Indigenous youth, who are considerably overrepresented (53.8%) within the Canadian foster care system [96], made up 36.8% of the sample. Approximately one quarter of respondents (25.7%) described themselves as LGBTQ + , compared to only 4% of the Canadian general population [97]. This elevated proportion mirrors findings of related North American studies, which show an overabundance of sexual- and gender-minority youth in child welfare systems [98–100]. Contributing factors may include family rejection, discrimination-related placement instability, and a greater sense of safety to disclose identity within affirming research contexts. Forty percent of participants were parents, and every province and territory was represented in the sample. While youth entered the foster care system for a variety of reasons, 74.4% were initially placed in care due to abuse or neglect and/or exposure to domestic violence. Participants spent an average of 9.4 (4.5 SD) years in foster care, with 43.5% having had five or more placements.

**Table 2:** Sociodemographic Characteristics.

Characteristic	All Participants (*n* = 203)		Interviewed Participants (*n* = 26)	
	Frequency (*n*) ^a^	Percentage (%) ^b^	Frequency (*n*) ^a^	Percentage (%) ^b^
*Mean Age (Years)* ^c^	23.6 (2.6) ^d^		24.4 (2.4) ^d^	
*Race* ^e^				
Black	52	27.1	6	24.0
East Asian	11	5.7	1	4.0
Latin American/Hispanic	30	15.6	0	0.0
Middle Eastern	12	6.3	0	0.0
South Asian	25	13.0	2	8.0
Southeast Asian	9	4.7	0	0.0
White	65	33.9	12	48.0
Other	8	4.2	4	16.0
*Indigenous*	70	36.8	7	26.9
*Gender*				
Woman	110	54.2	16	61.5
Man	80	39.4	5	19.2
Non-Binary	12	5.9	4	15.4
Other	1	0.5	1	3.9
*LGBTQ+*	48	25.7	11	44.0
*In a Relationship*	73	38.0	12	46.2
*Parenting*	78	40.0	7	26.9
*Disability*	41	23.7	10	40.0
*Education Level*				
Some High School	20	10.2	1	3.9
High School Diploma	52	26.4	2	7.8
Some Post-Secondary	45	22.8	7	26.9
College Diploma	43	21.8	8	30.8
Bachelor’s Degree	32	16.2	6	23.1
Master’s Degree	1	0.5	1	3.9
Other	4	2.0	1	3.89
*Employment Status*				
Working Full-Time	50	25.4	13	50.0
Working Part-Time/Per Diem	70	35.5	4	15.4
Unemployed	51	25.9	3	11.5
On Temporary Leave	16	8.1	1	3.9
Unable to Work	2	1.0	0	0.0
Other	8	4.1	5	19.2
*Job-Seeking*	103	51.8	13	50.0
*Annual Household Income*				
≤ $24,999	99	55.9	14	58.3
$25,000 – $49,999	52	29.4	5	20.8
$50,000 – $74,999	25	14.1	5	20.8
≥$75,000	1	0.6	0	0.0
*Income Supplement*	43	23.9	17	68.0

^a^Frequencies may not add to 203 if participants chose not to answer the question

^b^Percentages may not add to 100 if participants selected more than one response

^c^Continuous variable

^d^Numbers in parentheses are standard deviations

^e^Select all that apply

**Table 3:** Foster care characteristics.

Characteristic	All Participants (*n* = 203)		Interviewed Participants (*n* = 26)	
	Frequency (*n*) ^a^	Percentage (%) ^b^	Frequency (*n*) ^a^	Percentage (%) ^b^
*Reason for Initial Placement* ^e^				
Parental Death or Illness	55	27.6	14	53.9
Parental Incarceration	28	14.1	5	19.2
Exposure to Domestic Violence	68	34.2	16	61.5
Abuse or Neglect	80	40.2	20	76.9
Abandonment	46	23.1	11	42.3
Juvenile Offenses	23	11.6	0	0.0
Voluntary Placement	21	10.6	6	23.1
Other	7	3.5	2	7.7
*Mean Time in Care (Years)* ^c^	9.4 (4.5) ^d^		8.5 (5.0) ^d^	
*Total Number of Placements*				
1–2	56	29.3	6	25.0
3–4	52	27.2	4	16.7
≥ 5	83	43.5	14	58.3
*Placement Location(s)* ^e^				
British Columbia	25	12.6	5	19.2
Alberta	21	10.6	2	7.7
Saskatchewan	10	5.0	2	7.7
Manitoba	32	16.1	2	7.7
Ontario	59	29.7	16	61.5
Quebec	14	7.0	0	0.0
New Brunswick	6	3.0	0	0.0
Nova Scotia	8	4.0	1	3.9
Prince Edward Island	5	2.5	0	0.0
Newfoundland & Labrador	9	4.5	1	3.9
Yukon	4	2.0	0	0.0
Northwest Territories	11	5.5	0	0.0
Nunavut	4	2.0	0	0.0
*Transition Supports* ^e^				
Stipend or Financial Aid	70	36.5	12	46.2
Housing Assistance	61	31.8	3	11.5
Secondary Health Benefits	53	27.6	12	46.2
Mental Health Services	67	34.9	3	11.5
Education Grants	79	41.2	15	57.7
Life Skills/Employment Training	67	34.9	2	7.7
Transition Worker or Advocate	47	27.5	4	15.4

^a^Frequencies may not add to 203 if participants chose not to answer the question

^b^Percentages may not add to 100 if participants selected more than one response

^c^Continuous variable

^d^Numbers in parentheses are standard deviations

^e^Select all that apply

The interview subsample (*n* = 26) was slightly less heterogeneous, with nearly half (48%) of participants describing themselves as White, 61.5% as women and 53.9% as single. Forty-four percent considered themselves to be LGBTQ+ and 40% disclosed having a debilitating physical or mental health condition, self-defined as a disability. While participants in the interview subsample spent less time in care, on average (8.5 years, 5.0 SD), a higher proportion (58.3%) had five or more placements.

### Quantitative Findings

Descriptive statistics and reliability coefficients (alphas ranged 0.76 to 0.92) for each of the study instruments are presented in Table 4.

**Table 4:** Descriptive statistics for study instruments.

Instrument	Mean	SD	Minimum	Maximum	% Missing	Cronbach’s *α*
Benevolent Childhood Experiences [BCE] *n *= 203	6.3	2.5	0	10	0.0	0.76
Adverse Childhood Experiences [ACE] *n *= 203	3.7	2.6	0	10	0.0	0.81
Intersectional Discrimination Index (Anticipated) [InDI-A] *n *= 195	19.0	10.9	0	36	3.9	0.92
Intersectional Discrimination Index (Day-to-Day) [InDI-D] *n *= 203	6.1	2.6	0	9	0.0	0.84
Perceived Stress Scale [PSS] *n *= 170	22.2	6.0	8	38	16.3	0.81
PTSD Checklist for DSM-5 (8-Item) [PCL5–8] *n *= 180	16.4	7.4	0	32	11.3	0.87
PROMIS Emotional Distress-Anxiety (4-Item) [PROMIS-4a] *n *= 189	10.1	3.9	4	20	6.9	0.87
PROMIS Depression (6-Item) [PROMIS-6a] *n *= 165	13.8	5.6	6	30	18.7	0.92
Brief Resilience Scale [BRS] *n *= 198	3.1	0.8	1.00	4.83	2.5	0.80
Secure Flourishing Index [SFI] *n *= 145	70.3	22.8	5	116	28.6	0.89

*Note:* In the absence of a “yes” (1), missing values for the BCE, ACE, and InDI-D were scored as “no” (0)

The correlation matrix of binary associations between study variables is displayed in Table 5, with moderate to strong correlations (*r* ≥ 0.3) highlighted in grey and darker shades denoting stronger correlations. Each of the four structural violence variables was positively correlated with all three mental health variables. Anticipated intersectional discrimination and perceived stress were also inversely linked to positive adaptation variables, suggesting that higher InDI-A and PSS scores are associated with less resilience and flourishing. Mental health variables were negatively correlated with positive adaptation variables, indicating that greater PTSD, anxiety, and depression also correspond with lower BRS and SFI scores. Even though the correlation coefficient for anxiety and resilience was borderline moderate, these variables were considered as elements of mental health and positive adaptation, and so were seen as worthy of further exploration. Benevolent childhood experiences were weakly correlated with other constructs, so were not included in further regression models.

**Table 5:** Correlation Matrix for Study Variables.

Instrument	1.	2.	3.	4.	5.	6.	7.	8.	9.	10.
1. Benevolent Childhood Experiences *n *= 203 [Protective Factors]	1.00									
2. Adverse Childhood Experiences *n *= 203 [Structural Violence]	−0.23^b^	1.00								
3. Intersectional Discrimination Index (Anticipated) *n *= 194 [Structural Violence]	−0.35^d^	0.17^a^	1.00							
4. Intersectional Discrimination Index (Day-to-Day) *n *= 172 [Structural Violence]	−0.24^c^	0.40^d^	0.31^d^	1.00						
5. Perceived Stress Scale *n *= 170 [Structural Violence]	−0.29^c^	0.31^d^	0.46^d^	0.27^c^	1.00					
6. PTSD Checklist for DSM-5 (8-Item) *n *= 180 [Mental Health Challenges]	−0.27^c^	0.39^d^	0.48^d^	0.38^d^	0.73^d^	1.00				
7. PROMIS Emotional Distress-Anxiety (4-Item) *n *= 189 [Mental Health Challenges]	−0.23^b^	0.45^d^	0.35^d^	0.39^d^	0.69^d^	0.70^d^	1.00			
8. PROMIS Depression (6-Item) *n *= 165 [Mental Health Challenges]	−0.23^b^	0.45^d^	0.35^d^	0.40^d^	0.73^d^	0.67^d^	0.81^d^	1.00		
9. Brief Resilience Scale *n *= 198 [Positive Adaptation]	0.13	0.07	−0.33^d^	−0.12	−0.49^d^	−0.33^d^	−0.27^c^	−0.30^c^	1.00	
10. Secure Flourishing Index *n *= 145 [Positive Adaptation]	0.31^c^	−0.13	−0.42^d^	−0.20^a^	−0.42^d^	−0.36^d^	−0.39^d^	−0.50^d^	0.37^d^	1.00

^a^*p* < .05 ^b^ *p* < .01 ^c^ *p* < .001 ^d^ *p* < .0001

Moderate to strong correlations (r > ±0.3) are shaded

Ten block sequential multiple linear regression models tested relationships between individual outcome variables and larger quantitative constructs, as a set of predictors. For each outcome variable, six sociodemographic characteristic and foster care history covariates (i.e., age, time in care, Black racial group, Indigenous background, LGBTQ+ identity, and five or more placements) were applied to the model as a block. All variables belonging to each quantitative construct (e.g., resilience and flourishing, representative of positive adaptation) were then applied to the model as a second block to test the explanatory power of the quantitative construct on a single outcome variable. The selection of covariates and relationships tested were informed by theory and the correlation matrix.

Table 6 summarizes the Δ*R*^2^ and statistical significance for each relationship tested through regression modelling. These values represent the incremental increase in the model *R*^2^ resulting from the addition of a quantitative construct (structural violence, mental health, or positive adaptation) as a second block. Of the ten models (see supplementary Tables 1–9 in S1 File), three had a moderate to high Δ*R*^2^ (≥ 0.5), indicating that adding a given set of predictors to the model had a substantial effect on the outcome variable, beyond that explained by covariates. All Δ*R*^2^ values were significant at the < .01 or <.001 level.

**Table 6:** Summary of Δ*R*^2^ for All Regression Models.

		Outcome Variable (Dependent)				
		PCL8−5	PROMIS Anx. 4a	PROMIS Dep. 6a	BRS	SFI
**Quantitative Construct (Independent)**	*Structural Violence* ACE InDI-A InDI-D PSS	0.61***	0.57***	0.60***	0.28***	0.19***
	*Mental Health Challenges* PCL8−5 PROMIS Anx. 4a PROMIS Dep. 6a				0.11**	0.26***
	*Positive Adaptation* BRS SFI	0.20***	0.20***	0.36***		

Note: *p < .05, **p < .01, ***p < .001

Table 7 serves as an exemplar model, testing how much of the variance in PTSD levels (PCL8−5) is explained by structural violence, including adverse childhood experiences, anticipated and daily intersectional discrimination, and perceived stress as a combined set of predictors. For this model, Δ*R*^2 ^= 0.61 and *p* < .001, indicating that the full model explains significantly more of PTSD than covariates alone. Within this relationship, Indigenous background, LGBTQ+ identity, adverse childhood experiences, anticipated intersectional discrimination, and perceived stress were each significant predictors, when controlling for all other variables in the model.

**Table 7:** Block Sequential Regression Exemplar: PTSD, Predicted by Structural Violence and Sociodemographic Characteristics.

Predictor Variable	Model 1 (Sociodemographics Only)		Model 2 (w/ Structural Violence Variables)	
	Estimate (*b*)	95% CI	Estimate (*b*)	95% CI
Age (Years)	−0.24	−0.67, 0.19	−0.30	−0.60, 0.01
Time in Care (Years)	−0.05	−0.33, 0.22	0.06	−0.13, 0.26
Black Racial Group	1.97	−0.69, 4.64	1.30	−0.53, 3.12
Indigenous Background	1.29	−1.22, 3.80	2.19*	0.35, 4.03
LGBTQ+ Identity	0.32	−2.32, 2.96	−1.99*	−3.86, −0.12
5 or More Placements	2.28	−0.16, 4.72	−0.82	−2.64, 1.00
Adverse Childhood Experiences	**–**	**–**	0.65***	0.30, 1.00
Discrimination (Anticipated)	**–**	**–**	0.14**	0.05, 0.23
Discrimination (Day-to-Day)	–	–	0.33	−0.02, 0.69
Perceived Stress	**–**	**–**	0.67***	0.51, 0.82
*R* ^*2*^	0.05		0.65	
*R* ^*2*^_*adj*_	0.01		0.63	
Δ*R*^*2*^	–		0.61***	

Note: *p < .05, **p < .01, ***p < .001

### Qualitative findings

Nine themes were developed: (1) *Trauma Through Time*, (2) *Continued Chaos*, (3) *Searching for Stability*, (4) *Helpers and Havens*, (5) *Aging Out and Adulting*, (6) *Relationships and Responsibilities*, (7) *Health Challenges and Changes*, (8) *Remarkable Resilience*, and (9) *Moving Onward and Upward*. An overarching theme was conceived: *‘Escaping the trauma, building a life, changing the system’,* which reflects the complexity of this clinical issue, underscoring how the socioecological, temporal, and intersectional aspects of participants’ lived experiences converged to make meaning, and thus influence perceptions.

Fig 4 illustrates how we mapped each theme onto the study’s conceptual model. In this figure, the surface area of the theme (white) overlaps with all four social ecosystems (green) pictured, and also spans several developmental stages (red), extending from early life to adulting. An exemplar quotation from a single participant cuts across the concentric circles, representing how one’s intersectional identities (blue) influenced the ways in which they interacted with their social and built environment, ultimately affecting their guiding beliefs and worldview. Figures displaying the remaining themes are located in supplemental materials.

#### Trauma through time.

Trauma and violence shadowed participants before, during, and after their time in foster care. Early adverse experiences were found to be an indicator of traumatic exposure encountered upon aging out and entering adulthood. The compounding effects of such deep and layered trauma served to shape participants’ future relationships, personal identities, and mental health: “I definitely hit some rough patches. I think leaving foster care, you’re left with many open wounds” (Participant 12).

#### Continued chaos.

Participant 11 likened youth-in-care to “feral children not being taken care of”. Throughout childhood and adolescence, participants were tasked with navigating and balancing complex relationships with both their biological and foster families. A general sense of precarity, detachment, and abandonment led many participants to feel alone and disenfranchised. While predominantly negative, foster care placements were extremely polarizing, varying from rehabilitative to abusive. Ever-changing caseworkers, foster parents, and circumstances supported a constant and chaotic state of flux, with 14 participants having had five or more foster care placements**.**

#### Searching for stability.

Participants represented diverse identities, communities, and lived experiences, yet shared a common ‘foster kid’ status. Participants spoke of social isolation during childhood, as well as divergent behaviors in response to stressors (e.g., defiance vs. perfectionism). Many felt that the nature of their foster care experience—that is, whether it was positive or negative—was “just a circumstantial roll of the dice” (Participant 11). A sense of transience and instability was described by all participants (n = 26), with most moving frequently from placement to placement. Understandably, they craved a sense of belonging and a place to call home. Marginalizing identities affected participants’ interactions with the foster care system, and were associated with experiences of racism, discrimination, and stigma.

#### Helpers and havens.

While navigating the foster care system, myriad supports and services were available to participants; however, many participants lacked access to, or even awareness of, such essential resources. caseworkers (typically social workers) had powerful influence over participants’ individual trajectories: “I think that your social worker plays a big part in your success” (Participant 2). Whether due to positive or negative experiences, caseworkers influenced the career and community engagement choices of participants; many of whom were inspired to work with foster children or those aging out. Feeling as though “the system is terribly failing” (Participant 15), systemic challenges and barriers have motivated participants to become involved in advocacy efforts, with respect to Canadian foster care reform: “I’m fighting for what we went through. I’m fighting for more help, more assistance, more resources” (Participant 12).

#### Aging Out and Adulting.

The process of aging out, and the coinciding transition to adulthood, felt like a “big looming thing that’s over your head” (Participant 25). Filled with much anticipation, uncertainty, and apprehension, all participants (*n* = 26) voiced a general lack of control during this time. Many also described a sense of instability, amid the absence of supportive relationships. Aging out marked an abrupt and dramatic shift in participants’ lives. Seemingly overnight, participants were granted independence, freedom, and autonomy. They were also entrusted with greater responsibility and were expected to make complex decisions without assistance. Most participants reflected upon the experience as a meaningful opportunity for personal growth and development, even if the process itself was negative: “this is … where your life starts” (Participant 21). While this transition marked an exciting new era, many “felt extremely ill-equipped” (Participant 8) when aging out of care. Participants were left to navigate the complexities of emerging adulthood with minimal guidance and assistance, and were often unaware of their rights and available benefits.

Among the many issues discussed during interviews, (1) housing, (2) life skills, and (3) mentorship were most commonly raised by participants, with many describing unstable housing and periods of homelessness following emancipation. Participants reported “couch-surfing”, relying on friends or temporary accommodations, or exhausting transitional-housing supports after eligibility expired. These experiences were attributed to insufficient financial assistance, limited affordable housing options, and systemic barriers that disproportionately affect former youth-in-care. Housing instability was often intertwined with poor mental health, employment insecurity, and disruptions to education. Lacking learned skills concomitant with the adult role, such as cooking and budgeting, participants also expressed a keen interest in acquiring diverse knowledge and expertise that would assist in everyday situations. Further, participants craved formal mentorship by trusted adult role models who could serve as both a guide and confidant as youth approached the point of aging out, and continuing post-transition. There were varying opinions on when youth should age out of care, with the “ideal age” ranging from 18 to 30. When asked, all but one participant (*n* = 25) felt that youth should age out later than is currently mandated in their jurisdiction (typically 18 or 19), or at least be given the option to do so.

#### Relationships and responsibilities.

Participants sought safe and supportive relationships at every stage of their development, but had greater choice and control over those connections after aging out of care. Many spoke of building their own network of “chosen family,” friends, partners, and even pets that served as a support system and safety net: “I created my own family [tree]” (Participant 24). For some, continued or cyclic involvement in harmful relationships stemmed from a fear of being alone. Unfortunately, experiences of abuse and abandonment were all too common among participants with partners, and particularly those with children. Of the seven interview participants who were parents, all wanted a good life for their children and families, and in many cases hoped to provide a better future than they, themselves were offered. Participants demonstrated persistence and adaptability as they learned to navigate relationship and parenting responsibilities.

#### Health challenges and changes.

Participants discussed how past experiences of trauma and violence, embedded within toxic environments, laid the groundwork for various health challenges. Self-identified mental health symptoms and formally diagnosed conditions were pervasive, with every participant sharing at least one psychological complaint. Among the many symptoms described, issues related to mood dysregulation, anxiety, relational attachment, and grief were most prevalent. Several participants also shared devastating previous experiences of self-harm, suicidal ideation, and attempts. Other common mental health challenges included developmental disabilities, disordered eating behaviors, substance use, and alcohol dependence. Nearly all participants expressed frustration regarding the accessibility of mental health care post-transition. Participants referenced the prohibitively high out-of-pocket costs, lengthy wait times for counselors and specialists, and the abrupt loss of pediatric care upon aging out and entering young adulthood. It was also felt that mental health practitioners were untrained, under supported, and ill-equipped to meet the needs of former youth-in-care. Participants sought intensive trauma-focused therapy that was specifically tailored to those with foster care system involvement. When asked about their mental health directly, participants reported that their overall mental health was better than they had experienced in the past; however, most also described some variability depending upon the circumstances or context: “mental health is like, like it’s peaks and valleys” (Participant 8). Physically, most participants were in good health, with many engaging in health promotive lifestyle behaviors as a form of adaptive coping. Unfortunately, many faced difficulties finding an appropriate and consistent primary care provider. Of those available, very few clinicians had any knowledge of the foster care experience, and often applied harmful stereotypes to the provider-client relationship.

#### Remarkable resilience.

Despite facing incredible adversity throughout childhood, adolescence, and emerging adulthood, all participants demonstrated characteristics consistent with resilience; although some far more than others. While a few participants struggled to cope with life’s challenges, most were able to ‘bounce back’ in response to negative experiences. Participants carefully considered past experiences and thought deeply about their personal identities, beliefs, and behaviors. By exploring the past, participants were better able to make sense of their current life circumstances, as well as future goals and dreams: “if it wasn’t for my experience, I wouldn’t be where I am right now” (Participant 12). Through such introspection and reflection, many participants achieved personal growth: the process of “becoming a warrior” (Participant 1). Participants also used a wide variety of coping strategies and self-care practices to support their overall wellbeing. Many expressed gratitude and satisfaction with respect to their current life circumstances, and above all else, were thankful for the person they had become (irrespective of the trauma they had endured): “I’m like comfortable with who I am. I know who I am … I’m just more sure of myself” (Participant 17). Participants thrived when they had adequate support. This required most to build connections proactively through self-advocacy. Recognizing that “a closed mouth doesn’t get fed” (Participant 5), participants often had to ask for help explicitly in order to meet basic needs. Along their personal journeys toward growth and healing, many participants sought to bring about change through advocacy and altruism. Specifically, participants devoted time and effort to supporting current and former youth-in-care to “be who you needed when you were younger” (Participant 22). Participants looked forward to the future and set meaningful goals for themselves: “I feel like I’m more empowered than I have ever been, and I’m excited finally to see where life’s gonna take me” (Participant 24).

#### Moving Onward and Upward.

Participants spoke of the future, including hopes for themselves, their families, and the foster care system at-large. Many felt that the system was changing for the better, and planned to draw upon their own experiences when “fighting the good fight” (Participant 20) and advocating for continued improvements. However, some worried that their changemaking efforts may have been hindered by a lack of power and resources. Most participants simply desired freedom, calm, and a better life than they had growing up. There was a resounding preference for a low-stress living environment, featuring a kind and supportive community: “I’ve had enough drama to last me a lifetime. I’ve had enough, you know, uncertainty to last me a lifetime. I don’t need to go find myself or explore or something like that. I just … wanna chill” (Participant 5). Many wished to own a home, pursue higher education, travel, and sought healthy relationships with people who truly cared. Career goals were often altruistic in nature, with many participants wanting to work with current or former youth-in-care in some capacity. However, some feared the potential for emotional burden and re-traumatization concomitant with such a role. In achieving greater independence and self-sufficiency, participants wished for transitional housing programs, robust financial supports, comprehensive life skills training, and professional mentorship.

### Mixed methods integration

In order to gain a more holistic understanding of associations among structural violence, mental health challenges, and positive adaptation, all data were merged, and findings integrated. The greater part of the integration phase was spent *comparing* quantitative and qualitative data for the broader sample and identifying general convergent and divergent findings; and *matching* individual participants’ survey results with corresponding interview responses, and closely examining divergent cases. Findings at both the participant and sample level were concordant across all domains, indicating close alignment between quantitative and qualitative data.

Participant profiles were constructed based on an individualized blend of sociodemographic characteristics and foster care histories. Such groupings were then used to compare instrument scores and interview responses. For participants who identified as Black or Indigenous, anticipated and daily intersectional discrimination (InDI-A and InDI-D) scores were noticeably higher, suggesting that they anticipated and regularly faced more discrimination than their peers, on average. While this difference is at least partially attributable to racial or ethnic background, each of these participants embodied numerous other identities (e.g., LGBTQ+ status, education level, income) that may have moderated their experience of discrimination.

**Fig 5:**
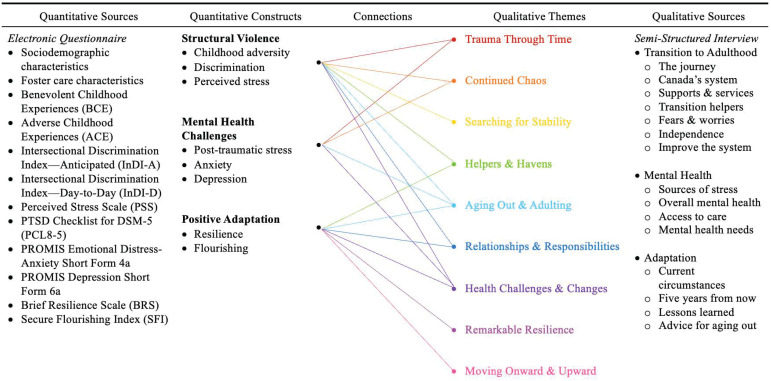
Linking Quantitative Constructs and Qualitative Themes.

Both *comparing* and *matching* strategies informed Fig 5, in which linkages between quantitative constructs and qualitative themes were made using color-coded connectors.

**Fig 6:**
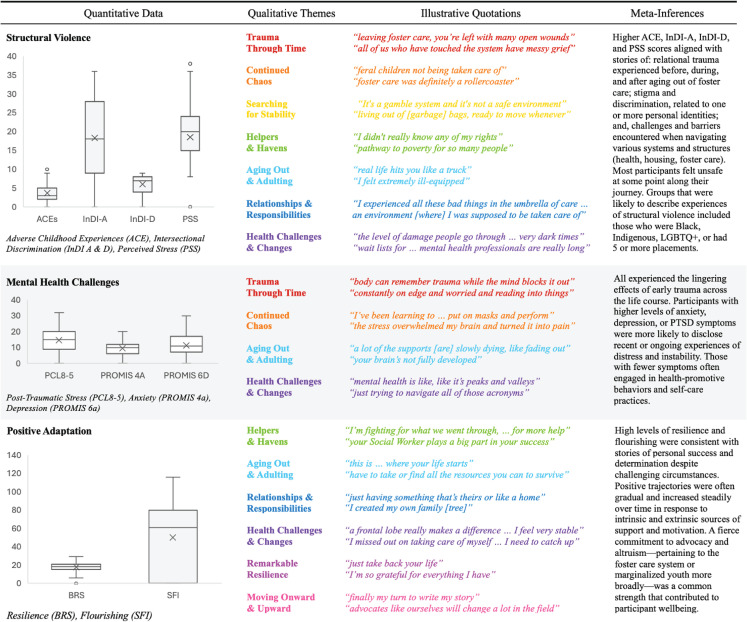
Traditional Joint Display. Note: Each box plot should be viewed in isolation, as scales and scoring practices varied across measures.

**Fig 7:**
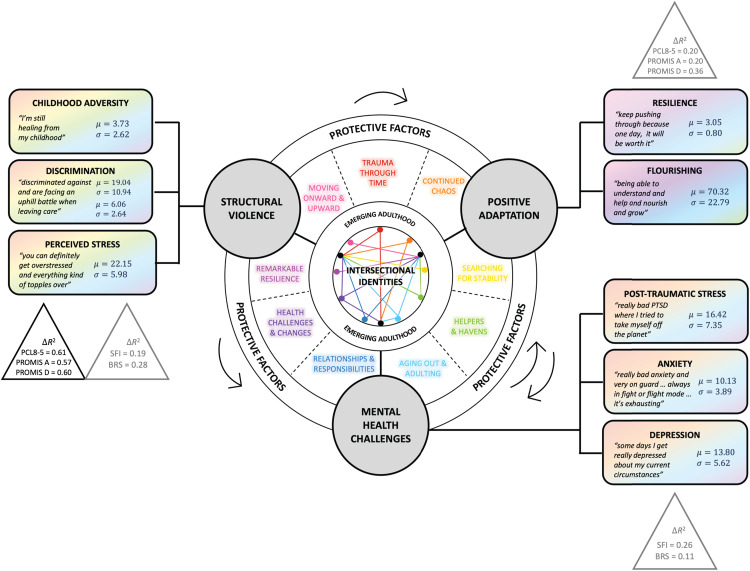
Comprehensive Joint Display.

Two joint displays (Figs 6 and 7) were created to illustrate the relationships depicted in Fig 5. For each of the three quantitative constructs—structural violence, mental health challenges, and positive adaptation, Fig 6 features quantitative data, qualitative themes, illustrative quotations, and meta-inferences. Box plots represent the distribution of instrument scores within the sample. Illustrative quotations for each connecting theme were selected as exemplars to bring qualitative findings to life, and meta-inferences were derived from the synergistic exchange of quantitative and qualitative insights. Structural violence variable scores corresponded with experiences of relational trauma, stigma and discrimination, and systemic challenges and barriers. Mental health challenges were linked to both current experiences of distress and instability, as well as the lingering effects of childhood trauma. Positive adaptation corresponded with stories of personal success and determination that increased steadily over time despite challenging circumstances.

Fig 7 graphically portrays the integration of mixed methods findings with study aims and the guiding conceptual model, by incorporating elements such as intersectional identities, emerging adulthood, and protective factors. Quantitative constructs and qualitative themes are prominently featured. Additionally, this joint display includes means and standard deviations for each instrument, quotations that align with each variable (rather than each theme), and changes in coefficients of determination (Δ*R*^2^) that correspond with the ten regression models used to analyze quantitative data.

The lingering effects of traumatic exposure were felt across the lifespan. Structural violence—whether experienced before, during, or after foster care—was associated with greater mental health challenges, and higher levels of anticipated intersectional discrimination and perceived stress were associated with less positive adaptation. By contrast, those with higher levels of positive adaptation exhibited fewer PTSD, anxiety, and depressive symptoms. Common positive adaptation characteristics included advocacy and altruism, with many participants endeavoring to influence positive change within the foster care system for the mutual benefit of themselves and others.

## Discussion

Using a convergent mixed methods approach [94], we investigated the intersection of structural violence, mental health challenges, and positive adaptation in emerging adults who aged out of foster care in Canada. Associations between such constructs existed within the broader context of trauma, violence, and marginalization, which not only affected how participants navigated their transition to independence, but also the personal meaning they ascribed to their experiences. Individual characteristics such as personality, appearance, abilities, and preferences also shaped reciprocal relationships between participants and their environment—both social and physical.

### Key findings

Key study findings represent overarching insights that stem from the integration of quantitative, qualitative, and mixed methods results. Here we discuss the study’s core constructs (structural violence, mental health challenges, and positive adaptation), as they relate to several supporting issues: intersectional identities, trauma history, protective factors, the transition to independence, and mental health care recommendations.

#### Structural violence and mental health.

Experiences of structural violence—encountered before, during, and after foster care—were associated with greater mental health challenges. Evidence of early adversity and continued traumatic exposure was captured through survey results and personal narratives, with participants reporting the many ways in which trauma and violence manifested across the lifespan. Through chronic activation of the ‘fight or flight’ stress response system [101], the lasting effects of emotional hardship were felt throughout the mind and body, both immediately and over time [102]. Participants endorsed high rates of PTSD, anxiety, and depressive symptoms, among other physical and mental health challenges, with many classifying their illness experience as a disability that interfered with daily functioning. Despite such clinical complaints, participants developed myriad self-preservation and defensive strategies to preserve their safety and wellbeing.

Having been repeatedly let down, or even harmed, by a system designed to protect them, participants acquired a dual sense of distrust and determination that helped them navigate the transition to independence. Despite such learned resourcefulness, participants were reliant upon various social services to meet their most basic needs, including safe and affordable housing. An objective lack of instrumental support, combined with experiences of discrimination, perceived stress, and complex trauma histories, led many participants to seek mental health care services as a means of coping with their past and current circumstances.

#### Structural Violence and Positive Adaptation.

Interestingly, the relationship between lived *experiences* of structural violence (as indicated by ACE and InDI-D scores) and positive adaptation was mixed. Direct comparisons of participant profiles suggested that for some, higher levels of adverse childhood experiences and daily intersectional discrimination were associated with lower levels of positive adaptation, while for others, structural violence was positively correlated with resilience and flourishing. Interview dialogue supported such divergent findings, with participants describing how adversities have either thwarted their success, or—perhaps less obviously—have served as a powerful motivator in their pursuit of ‘overcoming’ life’s hardships.

Conversely, *perceptions* of structural violence (InDI-A and PSS) were inversely associated with positive adaptation, suggesting that higher levels of anticipated intersectional discrimination and perceived stress were associated with less resilience and flourishing. This may indicate that participants’ worldview was more influential in determining their post-care trajectory than objective experiences of structural violence. Indeed, the meaning participants ascribed to past and present circumstances may have played an important role in their ability to navigate life’s challenges and stressors.

Common attributes associated with positive adaptation included advocacy, altruism, and self-care. Participants desired to influence positive change within the foster care system, and devoted their time and expertise to its sociopolitical advancement. Several volunteered with local community organizations, sat on advisory boards, participated in peer mentorship programs, and were involved in legal activism and policymaking efforts. Demonstrating a commitment to ‘giving back’, many also sought out opportunities to serve youth-in-care through careers such as social work. Some even voiced an interest in working for the child welfare system or becoming foster parents themselves. This interest in ‘helping the person behind you’ is a common finding across child welfare literature [103].

While such activities were outwardly focused and philanthropic in nature, participants disclosed that they derived great benefit from helping others. Participants’ involvement provided an opportunity to translate first-hand knowledge and experience into action [104]. Further, their leadership and community engagement contributed to a sense of empowerment and fulfillment conducive to resilience and flourishing. Those who exhibited high levels of positive adaptation typically benefitted from a robust social support network made up of ‘chosen family’ and trusted allies. Participants who were highly adaptive also engaged in a variety of health promotive behaviors, including physical activity, restorative sleep, recreational activities, personal reflection, and self-advocacy, among others. Such strategies mirror recommendations for same-aged peers, and are not unique to former youth-in-care [105].

#### Mental Health and Positive Adaptation.

Participants who exhibited higher levels of positive adaptation had fewer mental health challenges. While directionality was unclear, correlations and regression analyses demonstrated the inextricable linkage between these two constructs. Personal narratives bolstered such findings, with those exhibiting higher levels of resilience and flourishing less likely to report current symptoms of PTSD, anxiety, or depression; a finding consistent across studies and populations [106,107]. However, existing positive adaptation characteristics were not an indicator of past mental health status. Many participants shared stories of psychological distress and instability during adolescence, sometimes escalating to self-harm or suicidal ideation.

Further, an absence or lack of PTSD, anxiety, or depressive symptoms did not necessarily correspond with participants’ current mental health status—not only because measures focused solely on very recent experiences (within the past week to month) and may not have been a true reflection of more generalized attitudes and beliefs, but also because mental health is multifaceted [108], and mood, quality of life, and daily functioning can be affected in ways that do not align with traditional assessment tools. Indeed, participants’ self-appraisal of their mental health status was often a closer reflection of their positive adaptation level than objective measures of symptomatology, as determined through case constructing (participant profiles).

#### Intersectional Identities.

Compounding intersectional identities influenced how participants interacted with services and supports. Participants’ unique attributes not only affected how they perceived other people, places, and systems, but also how they were treated by those in positions of social, economic, or informational power [50]. Indeed, access to resources and opportunities was often influenced by institutional gatekeepers, who must have possessed the requisite knowledge, skill, and motivation to take appropriate action. Far too often, participants ‘missed out’ because they were not made aware or because they were told ‘no’ by caseworkers, Foster parents, and others. Owing to their unique background, some participants faced additional barriers to their success. For example, those who were Black or Indigenous frequently spoke about the challenges associated with foster care placements that were not culturally congruent [109].

It is also important to recognize that while not all identities were externally visible, they may have still influenced participants’ connections with the world, by guiding their thoughts, feelings, and behaviors, or by influencing their sense of safety and comfort [110]. The act of trying to conceal one’s identity when accessing services and supports may have also been distressing. When developing Fig 5, intersectional identities was removed as a quantitative construct because it was linked to all nine themes and overwhelmed the table. Indeed, intersectional identities are omnipresent and influence all interactions [50].

#### Trauma History.

When completing the Adverse Childhood Experiences (ACE) questionnaire, respondents frequently responded affirmatively to questions about the loss of a parent, living with someone who was mentally ill, and verbal abuse by a parental figure. The mean ACE score was 3.73 (*SD* = 2.62) out of 10, enough to pose a significant threat to physical and mental wellbeing [111], but just below the high-risk threshold for toxic stress [112]. These questions closely aligned with the top three reasons participants were initially placed into care: abuse or neglect, exposure to domestic violence, and parental death or illness, and were supported by descriptions of trauma and violence that continued as they entered the foster care system.

#### Protective Factors.

Several factors were protective, facilitating positive adaptation and guarding against mental health challenges. Of the items on the Benevolent Childhood Experiences (BCE) checklist, having at least one safe caregiver, comforting beliefs, and opportunities to have a good time were the most commonly selected responses. The mean BCE score was 6.27 (*SD* = 2.46) out of a possible 10. This total falls slightly below the average for studies involving comparable young adult populations [113], indicating that former youth-in-care may possess fewer protective factors than their peers. While this modest difference suggests limited clinical significance, it may nonetheless indicate the presence of specific protective factors that warrant further exploration.

Perhaps most influential, caseworkers were cited as ‘making or breaking’ the foster care experience. A strong caseworker maintained a professional, yet therapeutic, relationship and offered knowledge about the foster care system, including available resources and opportunities. They possessed authentic interpersonal skills and genuinely cared about participants’ success. Similarly, good foster parents served as positive role models committed to guiding, teaching, and providing for their foster children. Those who gave emotional, informational, and instrumental support as participants aged out (e.g., apartment hunting) were particularly valued. Emotional support encompasses empathy, care, and belonging; informational support includes guidance and advice; and instrumental support refers to tangible aid such as housing or financial assistance [114]. Participants described significant deficits across all three domains, underscoring the importance of holistic support systems. These findings align with a growing body of evidence on the capacity of a single adult caregiver—who provides diverse and comprehensive support— to buffer the effects of childhood trauma and adversity [115].

Participants expressed tremendous gratitude for the opportunity to take part in recreational activities, such as team sports leagues or summer camp. Such experiences gave participants the chance to ‘just be kids,’ and briefly set aside the stressors and worries associated with life as youth-in-care. For many, these served as highlights of participants’ childhood and adolescence. Among those who chose to pursue higher education, financial aid and tuition assistance programs offered by the provincial/territorial government afforded some participants the opportunity to attend college or university at little to no cost [116]. This supported their growth and development with respect to social networking, knowledge generation, and exposure to diverse perspectives.

#### Transition to Independence.

When aging out, participants encountered many systemic challenges and barriers that negatively affected their transition to adulthood. This finding closely aligns with that of other studies involving this population [4]. Time and time again, participants spoke of frustrations related to housing insecurity, financial hardship, and a lack of adult mentorship. Specifically, the pipeline from foster care to homelessness [117] was discussed at length. Without safe and affordable housing, several participants became street-involved, with others resorting to couch surfing or renting tiny, unkempt apartments in dangerous neighborhoods. While this was certainly a problem in its own right, poor (or absent) housing conditions were also credited with jeopardizing the most basic of needs, such as nutrition and hygiene, which in turn may have compromised other important dimensions of daily living, including education, employment, and personal relationships. Housing insecurity also promoted access to unsafe and illicit activities, including substance use and street crime. Indeed, interviews featured conversations about overdose, sexual assault, theft, and gun violence. Such findings lend support for a ‘housing first’ philosophy, which views housing as a pre-requisite for life improvement [118].

Financial strain served to restrict participants’ choices and freedoms. A modest living stipend seldom covered much beyond the cost of basic necessities, leaving participants to actively seek out additional sources of income. For some, this meant having to choose between education and full-time employment, while others worked tirelessly to strike a balance between both priorities. By contrast, same-aged peers from more ‘traditional’ households often received financial support that allowed them to focus solely on their studies and engage in opportunities for self-exploration, including participation in extracurricular activities and independent ventures such as travel. Participants also desired mentorship and guidance from a trusted adult role model, as well as basic life skills preparation. Many lacked confidence in basic activities associated with adulthood and independent living, such as cooking, budgeting, and home maintenance. Despite mandatory financial literacy classes offered in some jurisdictions, participants felt ill-prepared to manage their finances self-sufficiently.

Participants offered several systems-level recommendations that addressed the abovementioned barriers and facilitators to their wellbeing. They wanted individualized transition plans based on their unique strengths and needs, rather than a generic checklist. Another common request was for earlier and more robust preparation for the aging out process, encompassing close collaboration with a caseworker, practical transition assistance and life skills training (e.g., securing housing, establishing a personal budget, applying for post-secondary school), as well as activities to support emotional readiness. Many advocated for transitional housing specifically, identifying homelessness and housing instability as one of the most critical issues facing youth upon aging out of care [119]. For most, exiting the foster care system and entering emerging adulthood rendered participants ineligible for established pediatric supports. The continued provision or coverage of important and widely accessed services, such as dental care or therapeutic counselling, was also recommended. It should be noted that in recent years, some jurisdictions have made strides toward more comprehensive after-care benefits programs [120].

#### Mental health recommendations.

Participants were very appreciative of the system-specific mental health services they received while in foster care. Unfortunately, these were often not made known or available to all youth-in-care; rather, they were offered to the ‘most serious’ cases, such as those who had experienced psychosis or attempted suicide. Among participants who did receive such care, many wished that it would continue beyond the point of aging out—particularly with respect to prescribing and counseling services. Instead, services were typically discontinued abruptly without transition planning or a formal referral process. Further, former youth-in-care often fell to the bottom of wait lists for adult mental health services following their 18^th^ or 19^th^ birthday. During interviews, participants discussed a variety of barriers that interfered with their efforts to obtain psychological support as an adult, including prohibitive out-of-pocket costs, lengthy wait times, and inconsistent providers even when the need for care was urgent.

Many also had difficulty finding a provider who was the right ‘fit.’ Participants listed empathy, active listening, compassion, and lived experience as a foster child as desirable qualities that might improve their overall mental health care experience. Participants also recommended that psychiatrists, therapists, and others should receive dedicated training that addresses some of the unique strengths (e.g., courage, resourcefulness, self-advocacy), challenges (e.g., limited guidance and financial resources), and mental health needs (e.g., trauma- and violence-informed counselling) of former youth-in-care. It was felt that such education may also help to reduce healthcare provider stigma toward the ‘foster kid’ identity label. Participants desired intensive trauma- and violence-informed (TVI) therapy that focused on deep-seeded trauma, not just superficial or transient issues. Additionally, participants looked for providers who were skilled prescribers, as many experienced challenges related to medication use, such as finding the right formulation or guarding against substance use/addiction. For those who received inpatient psychiatric services, negative interactions with officers in uniform, such as police or security personnel, during the management of high-risk incidents were often reported. Participants recommended that officers be dressed in street clothing when present in mental health care settings to prevent re-traumatization.

### Strengths and Limitations

Strengths of this study included its conceptual and theoretical underpinnings, clinical and social relevance, study design, and sample size and diversity. This study addressed a significant gap in the literature, and has application to an important, highly vulnerable population that lacks relevant structured supports. The unification of three theoretical frameworks provided a unique lens through which to explore the phenomenon of interest, while the convergent mixed methods design supported a comprehensive understanding and the generation of meta-inferences through integration of quantitative and qualitative findings.

Young people aging out of foster care have traditionally been hard-to-reach, so an expansive social media strategy and targeted referrals through community agencies facilitated numerous connections, and the virtual platform allowed for convenience and facilitated widespread and diverse participant enrollment, spanning all provinces and territories across Canada. These efforts resulted in very diverse survey and interview participant samples, especially representing young people who are traditionally marginalized, and the moderately sized quantitative sample and findings from this exploratory study helps to strengthen implications and suggests directions for future research.

Study limitations included the study’s exploratory, cross-sectional design, measurement challenges, selection bias, and technology as a potential barrier to participation. The cross-sectional nature of this study prevented the examination of causal relationships between and among variables, and the exploratory approach prevented hypothesis-testing. Additionally, missing data resulted in inconsistent sample sizes. It was not possible to report construct validity for each measure, as comprehensive psychometric evaluation linking selected instruments to their corresponding theoretical concepts (structural violence, mental health, positive adaptation, and protective factors) has not been conducted in this specific population, and was not assessed as part of this study. A lack of national data pertaining to Canada’s former youth-in-care prevented the random selection of participants. Purposive sampling through community organizations and social media may have introduced selection bias.

The use of technology for data collection purposes may have introduced bias into the sample by excluding otherwise-eligible youth who did not have an electronic device, or did not have access to social media recruitment posts. However, potential participants were encouraged to access a freely available computer through a local community organization, and efforts to remain in contact with participants throughout the study via email, phone, or text helped to strengthen communication and promote continued involvement.

### Implications

#### Research and Scholarship.

The exploratory nature of this study facilitated the dissemination of novel findings and insights, and served to assess feasibility with respect to recruitment strategies, procedural safeguards, and virtual data collection methods. With scant descriptive data pertaining to Canada’s current or former youth-in-care, our success regarding cross-country recruitment validates the need and feasibility for large scale, nationally representative data collection efforts that reflect the diversity and experiences of Canadian emerging adults aging out of foster care. Grounded within an emancipatory paradigm, and theory-driven conceptual framework, the study design and findings may also encourage the adoption of equity- and justice-oriented research approaches that promote participant empowerment. Knowledge mobilization strategies that incorporate participant perspectives and leverage existing advocacy efforts may facilitate awareness and understanding of key study findings among current and former youth-in-care and their allies, as well as those in positions of power and influence. Future research may more wholly integrate participatory and community-engaged methods.

Based on study findings, we recommend further investigation regarding mental health care service delivery gaps encountered upon aging out of care, and research that focuses specifically on the unique experiences and needs of former youth-in-care with a second marginalizing identity (e.g., Indigenous, LGBTQ + , disability). Studies that explore the research problem from alternative viewpoints—by describing the perspectives of caseworkers, foster parents, or healthcare providers, for example—may help to provide a more comprehensive understanding of the challenges faced by this population, and additional opportunities for action. Greater exploration of protective factors may also support the development of health-promotive interventions. Striking qualitative findings suggest the need for quantitative analysis involving the examination of individual items contained within the Benevolent Childhood Experiences (BCEs) questionnaire, which closely aligned with participants’ interview responses. Issues such as the presence of a safe caregiver and opportunities to have fun may serve as the basis for further study.

#### Health and Social Policy.

Study findings have the potential to inform upstream policies and procedures that address sources of structural violence, as opposed to reactive approaches that target its negative consequences. Evidence of varied participant transition experiences and trajectories lends credibility to the growing national push for flexible emancipation policies that are based on ‘readiness,’ rather than chronological age. Building off the sample diversity achieved in this study, continued efforts to recruit participants from across Canada may illuminate legislative differences by jurisdiction, thus highlighting regional strengths and shortcomings, and encouraging the cross pollination of innovative ideas.

Participants voiced an interest in policies that would support expanded access to mental health care upon aging out. Dedicated mental health providers assigned to former youth-in-care transitioning to independence would help to alleviate some of the fears and anxieties that accompany emancipation. Full coverage for psychiatric and counselling services would also lessen the financial burden that is a barrier to both preventative and rehabilitative mental health care. Many participants also commented on the enormity of their caseworkers’ caseloads, making it challenging for them to devote the necessary time, energy, and attention to each foster child on their roster. This was particularly problematic for youth actively preparing to age out of care, as they required individualized administrative support. Policies that cap caseloads and stipulate target client ratios may reduce caseworker fatigue and burnout, and encourage more helpful and supportive relationships.

Transitional housing was raised time and time again as an innovative solution to housing insecurity and homelessness among former youth-in-care. While the provision of free or subsidized physical accommodations would undoubtedly be ideal, policies that mandate child welfare agencies to seek out, secure, and approve housing options for youth aging out would help to reduce stress and enhance their health and safety. Other youth-championed policy recommendations that should be considered include consistently placing family members (particularly siblings) together, offering a tuition waiver for post-secondary education, and implementing mandatory life skills training with measurable objectives for foster children and their foster parents. While certain provinces/territories may have versions of these policies, they have not been implemented in most jurisdictions nationally.

#### Clinical Practice.

Findings illuminated unique challenges and stressors faced by this population when transitioning to independence, as well as common characteristics and circumstances associated with positive adaptation. Such evidence may support the development and testing of tailored TVI mental health care interventions for youth aging out of care, and contribute to greater clinical awareness of their unique mental and physical health needs. Lessons learned may extend beyond the foster care domain, highlighting important care considerations for other ‘high risk’ groups of youth (e.g., Indigenous, justice-involved, or unhoused).

Findings from this study suggest that the health care system is mostly ill-equipped to meet the physical and mental health needs of former youth-in-care. Participants desired health professionals, who were knowledgeable about the child welfare system. Unfortunately, most participants had grown accustomed to educating their clinicians about foster care, and often had to deal with harmful stereotypes and stigmatizing language. A mandatory online interdisciplinary clinical education module could improve understanding of the foster care system and the unique challenges faced by former youth-in-care, and may also enhance empathy toward such patients. Participants also shared that their foster parents were woefully unprepared to support them through mental health challenges and crises. Given the high prevalence of mental illness among foster youth [121], participants recommended that foster parents be required to complete comprehensive mental health training as part of their onboarding.

Qualitative interviews revealed an overwhelming commitment to advocacy and altruism as forms of positive adaptation. Participants were eager to ‘give back’ to current or former youth-in-care by offering their knowledge, experience, and support as professionals (e.g., social workers), volunteers, or potential foster parents. This dedication to bettering the foster care system and its youth can be channeled into robust clinical interventions that draw on community engagement and service to others (e.g., peer mentorship) to enhance mutual wellbeing. Such strategies have been shown to improve mental and physical health and longevity in a variety of populations [122].

## Conclusion

Given a lack of research in this field, the overall complexity of the research problem, and the limited clinical knowledge pertaining to this population, our integration of quantitative and qualitative findings led to a richer and more comprehensive understanding than would have been possible using either approach in isolation. Combining quantitative and qualitative data ensured that statistical findings were grounded in participants’ experiences, and that contextual nuances were aptly captured. This holistic appreciation, conducive to the discovery of overarching patterns and relationships, provides the groundwork for future follow-up study, and may also influence practical solution-driven action and intervention. Findings from this exploratory study may meaningfully contribute to the health and welfare of emerging adults aging out of foster care, lending support for continued exploration of this complex and understudied phenomenon.

## Supporting information

S1 File Quantitative and Qualitative Findings. Block sequential regression tables and qualitative thematic diagrams.
(PDF)
