# Supplementary material for: “Finally my turn to write my story”: A convergent mixed methods study exploring the perceptions and experiences of emerging adults who aged out of foster care in Canada
Source: PLoS One. 2025 Dec 29;20(12):e0338574. doi: 10.1371/journal.pone.0338574 (PMC12747405; doi:10.1371/journal.pone.0338574)

Appendix

Supplemental Table 1 Block Sequential Regression: Anxiety, Predicted by Structural Violence and Sociodemographic Characteristics

Predictor Variable	Model 1 (Sociodemographics Only)		Model 2 (w/ Structural Violence Variables)	
	Estimate	95% CI	Estimate	95% CI
Age (Years)	-0.01	-0.23, 0.22	0.04	-0.13, 0.20
Time in Care (Years)	-0.04	-0.19, 0.10	0.01	-0.10, 0.12
Black Racial Group	-0.24	-1.69, 1.22	-0.51	-1.56, 0.53
Indigenous Background	-1.15	-2.49, 0.19	-0.82	-1.84, 0.21
LGBTQ+ Identity	0.74	-0.64, 2.12	-0.06	-1.09, 0.97
5 or More Placements	0.45	-0.84, 1.73	-0.69	-1.69, 0.31
Adverse Childhood Experiences	-	-	**0.30****	0.11, 0.50
Discrimination (Anticipated)	-	-	0.04	-0.01, 0.09
Discrimination (Day-to-Day)	-	-	0.18	-0.02, 0.37
Perceived Stress	-	-	**0.38*****	0.30, 0.47
R^2	0.03		0.60	
R^2_{adj}	-0.01		0.57	
ΔR^2	-		**0.57*****	

Note: $*p < .05$, $**p < .01$, $***p < .001$

Supplemental Table 2 Block Sequential Regression: Depression, Predicted by Structural Violence and Sociodemographic Characteristics

Predictor Variable	Model 1 (Sociodemographics Only)		Model 2 (w/ Structural Violence Variables)	
	Estimate	95% CI	Estimate	95% CI
Age (Years)	0.17	-0.17, 0.52	0.13	-0.12, 0.38
Time in Care (Years)	-0.08	-0.31, 0.15	-0.05	-0.21, 0.12
Black Racial Group	-0.74	-2.90, 1.40	-0.55	-2.04, 0.95
Indigenous Background	-1.11	-3.24, 1.02	0.41	-1.16, 1.98
LGBTQ+ Identity	1.09	-1.02, 3.21	-0.40	-1.96, 1.16
5 or More Placements	0.91	-1.10, 2.91	-0.32	-1.86, 1.22
Adverse Childhood Experiences	**-**	**-**	**0.41****	0.12, 0.70
Discrimination (Anticipated)	**-**	**-**	0.01	-0.07, 0.09
Discrimination (Day-to-Day)	-	-	**0.36***	0.05, 0.67
Perceived Stress	**-**	**-**	**0.61*****	0.48, 0.73
R^2	0.03		0.63	
R^2_{adj}	-0.01		0.60	
ΔR^2	-		**0.60*****	

Note: *p <.05, **p < .01, ***p <.001

Supplemental Table 3 Block Sequential Regression: Resilience, Predicted by Structural Violence and Sociodemographic Characteristics

Predictor Variable	Model 1 (Sociodemographics Only)		Model 2 (w/ Structural Violence Variables)	
	Estimate	95% CI	Estimate	95% CI
Age (Years)	0.01	-0.03, 0.06	-0.01	-0.05, 0.04
Time in Care (Years)	-0.01	-0.04, 0.02	-0.01	-0.04, 0.01
Black Racial Group	-0.13	-0.40, 0.15	-0.11	-0.38, 0.16
Indigenous Background	0.01	-0.24, 0.27	0.18	-0.09, 0.45
LGBTQ+ Identity	0.26	-0.01, 0.53	0.20	-0.08, 0.48
5 or More Placements	**-0.33***	-0.58, -0.08	-0.16	-0.43, 0.11
Adverse Childhood Experiences	**-**	**-**	**0.08****	0.03, 0.13
Discrimination (Anticipated)	**-**	**-**	-0.01	-0.02, <0.01
Discrimination (Day-to-Day)	-	-	-0.02	-0.07, 0.03
Perceived Stress	**-**	**-**	**-0.07*****	-0.10, -0.05
R^2	0.07		0.35	
R^2_{adj}	0.04		0.30	
ΔR^2	-		**0.28*****	

Note: *p <.05, **p < .01, ***p <.001

Supplemental Table 4 Block Sequential Regression: Flourishing, Predicted by Structural Violence and Sociodemographic Characteristics

Predictor Variable	Model 1 (Sociodemographics Only)		Model 2 (w/ Structural Violence Variables)	
	Estimate	95% CI	Estimate	95% CI
Age (Years)	**2.43*** **	1.07, 3.78	**1.84** **	0.51, 3.17
Time in Care (Years)	-0.19	-1.10, 0.72	-0.01	-0.91, 0.90
Black Racial Group	-4.83	-13.20, 3.54	-6.90	-14.84, 1.03
Indigenous Background	0.42	-7.70, 8.53	2.36	-6.01, 10.73
LGBTQ+ Identity	1.02	-7.49, 9.54	1.13	-7.30, 9.57
5 or More Placements	**-16.02*** **	-24.18, -7.86	**-11.85** **	-20.30, -3.40
Adverse Childhood Experiences	-	-	-0.27	-1.87, 1.32
Discrimination (Anticipated)	-	-	-0.36	-0.78, 0.07
Discrimination (Day-to-Day)	-	-	-0.78	-2.37, 0.80
Perceived Stress	-	-	**-1.27*** **	-2.00, -0.55
R^2	0.19		0.38	
R^2_{adj}	0.15		0.32	
ΔR^2	-		**0.19*** **	

Note: *p <.05, **p < .01, ***p <.001

Supplemental Table 5 Block Sequential Regression: PTSD, Predicted by Positive Adaptation and Sociodemographic Characteristics

Predictor Variable	Model 1 (Sociodemographics Only)		Model 2 (w/ Positive Adaptation Variables)	
	Estimate	95% CI	Estimate	95% CI
Age (Years)	-0.24	-0.67, 0.19	0.10	-0.35, 0.55
Time in Care (Years)	-0.05	-0.33, 0.23	0.15	-0.14, 0.45
Black Racial Group	1.97	-0.69, 4.64	0.29	-2.35, 2.92
Indigenous Background	1.29	-1.22, 3.80	**3.09***	0.57, 5.61
LGBTQ+ Identity	0.32	-2.32, 2.96	0.79	-1.85, 3.43
5 or More Placements	2.28	-0.16, 4.72	-0.82	-3.50, 1.87
Brief Resilience Scale	-	-	**-2.12****	-3.65, -0.60
Secure Flourishing Index	-	-	**-0.08****	-0.14, -0.02
R^2	0.05		0.25	
R^2_{adj}	0.01		0.20	
ΔR^2	-		**0.20*****	

Note: *p <.05, **p < .01, ***p <.001

Supplemental Table 6 Block Sequential Regression: Anxiety, Predicted by Positive Adaptation and Sociodemographic Characteristics

Predictor Variable	Model 1 (Sociodemographics Only)		Model 2 (w/ Positive Adaptation Variables)	
	Estimate	95% CI	Estimate	95% CI
Age (Years)	-0.01	-0.23, 0.22	0.11	-0.11, 0.34
Time in Care (Years)	-0.04	-0.19, 0.10	0.03	-0.12, 0.17
Black Racial Group	-0.24	-1.69, 1.22	-0.67	-2.01, 0.67
Indigenous Background	-1.15	-2.49, 0.19	0.03	-1.25, 1.31
LGBTQ+ Identity	0.74	-0.64, 2.12	0.90	-0.24, 2.23
5 or More Placements	0.45	-0.84, 1.73	**-1.60***	-2.90, -0.20
Brief Resilience Scale	-	-	-0.71	-1.49, 0.06
Secure Flourishing Index	-	-	**-0.10*****	-0.09, -0.04
R^2	0.03		0.23	
R^2_{adj}	-0.01		0.18	
ΔR^2	-		**0.20*****	

Note: *p <.05, **p < .01, ***p <.001

Supplemental Table 7 Block Sequential Regression: Depression, Predicted by Positive Adaptation and Sociodemographic Characteristics

Predictor Variable	Model 1 (Sociodemographics Only)		Model 2 (w/ Positive Adaptation Variables)	
	Estimate	95% CI	Estimate	95% CI
Age (Years)	0.17	-0.17, 0.52	**0.39***	0.08, 0.69
Time in Care (Years)	-0.08	-0.31, 0.15	0.06	-0.13, 0.26
Black Racial Group	-0.74	-2.86, 1.38	-1.62	-3.34, 0.11
Indigenous Background	-1.11	-3.24, 1.02	1.34	-0.47, 3.15
LGBTQ+ Identity	1.09	-1.02, 3.21	0.80	-0.96, 2.57
5 or More Placements	0.91	-1.10, 2.91	**-2.25***	-4.08, -0.42
Brief Resilience Scale	-	-	**-1.35***	-2.42, -0.27
Secure Flourishing Index	-	-	**-0.12*****	-0.16, -0.08
R^2	0.03		0.39	
R^2_{adj}	-0.01		0.35	
ΔR^2	-		**0.36*****	

Note: *p <.05, **p < .01, ***p <.001

Supplemental Table 8 Block Sequential Regression: Resilience, Predicted by Mental Health and Sociodemographic Characteristics

Predictor Variable	Model 1 (Sociodemographics Only)		Model 2 (w/ Mental Health Variables)	
	Estimate	95% CI	Estimate	95% CI
Age (Years)	0.01	-0.03, 0.06	<0.01	-0.05, 0.06
Time in Care (Years)	-0.01	-0.04, 0.02	-0.02	-0.06, 0.02
Black Racial Group	-0.13	-0.40, 0.15	-0.21	-0.45, 0.13
Indigenous Background	0.01	-0.24, 0.27	0.07	-0.27, 0.40
LGBTQ+ Identity	0.26	-0.01, 0.53	-0.23	-0.09, 0.56
5 or More Placements	**-0.33***	-0.58, -0.08	**-0.32***	-0.63, <-0.01
PTSD Checklist for DSM-5	-	-	-0.01	-0.04, 0.01
PROMIS Anxiety	-	-	-0.02	-0.09, 0.05
PROMIS Depression	-	-	-0.02	-0.07, -0.02
R^2	0.07		0.18	
R^2_{adj}	0.04		0.12	
ΔR^2	-		**0.11****	

Note: $^*p$ <.05, $^{**}p$ < .01, $^{***}p$ <.001

Supplemental Table 9 Block Sequential Regression: Flourishing, Predicted by Mental Health and Sociodemographic Characteristics

Predictor Variable	Model 1 (Sociodemographics Only)		Model 2 (w/ Mental Health Variables)	
	Estimate	95% CI	Estimate	95% CI
Age (Years)	**2.43*** **	1.07, 3.78	**2.21** **	0.88, 3.54
Time in Care (Years)	-0.19	-1.10, 0.72	-0.08	-0.95, 0.79
Black Racial Group	-4.83	-13.20, 3.54	-5.27	-12.91, 2.38
Indigenous Background	0.42	-7.69, 8.53	4.33	-3.94, 12.59
LGBTQ+ Identity	1.02	-7.49, 9.54	-0.99	-8.73, 6.74
5 or More Placements	**-16.02*** **	-24.18, -7.86	**-14.08*** **	-22.00, -6.18
PTSD Checklist for DSM-5	-	-	-0.05	-0.76, 0.66
PROMIS Anxiety	-	-	-0.43	-2.20, 1.30
PROMIS Depression	-	-	**-2.05*** **	-3.14, -0.96
R^2	0.19		0.45	
R^2_{adj}	-0.15		0.40	
ΔR^2	-		**0.26*** **	

Note: **p* <.05, ***p* < .01, ****p* <.001

Supplemental Figure 1 Qualitative Theme: Continued Chaos

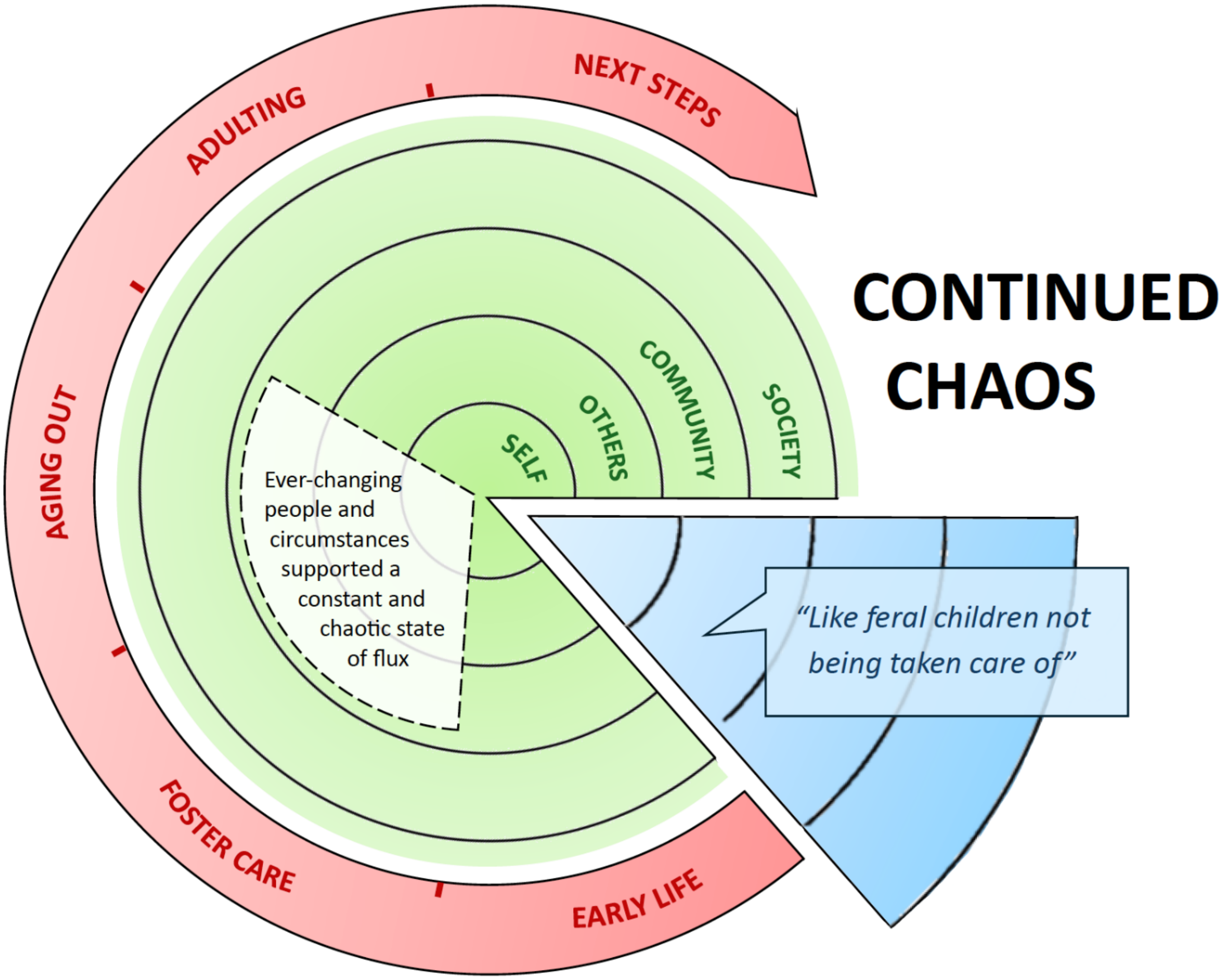

Supplemental Figure 2 Qualitative Theme: Searching for Stability

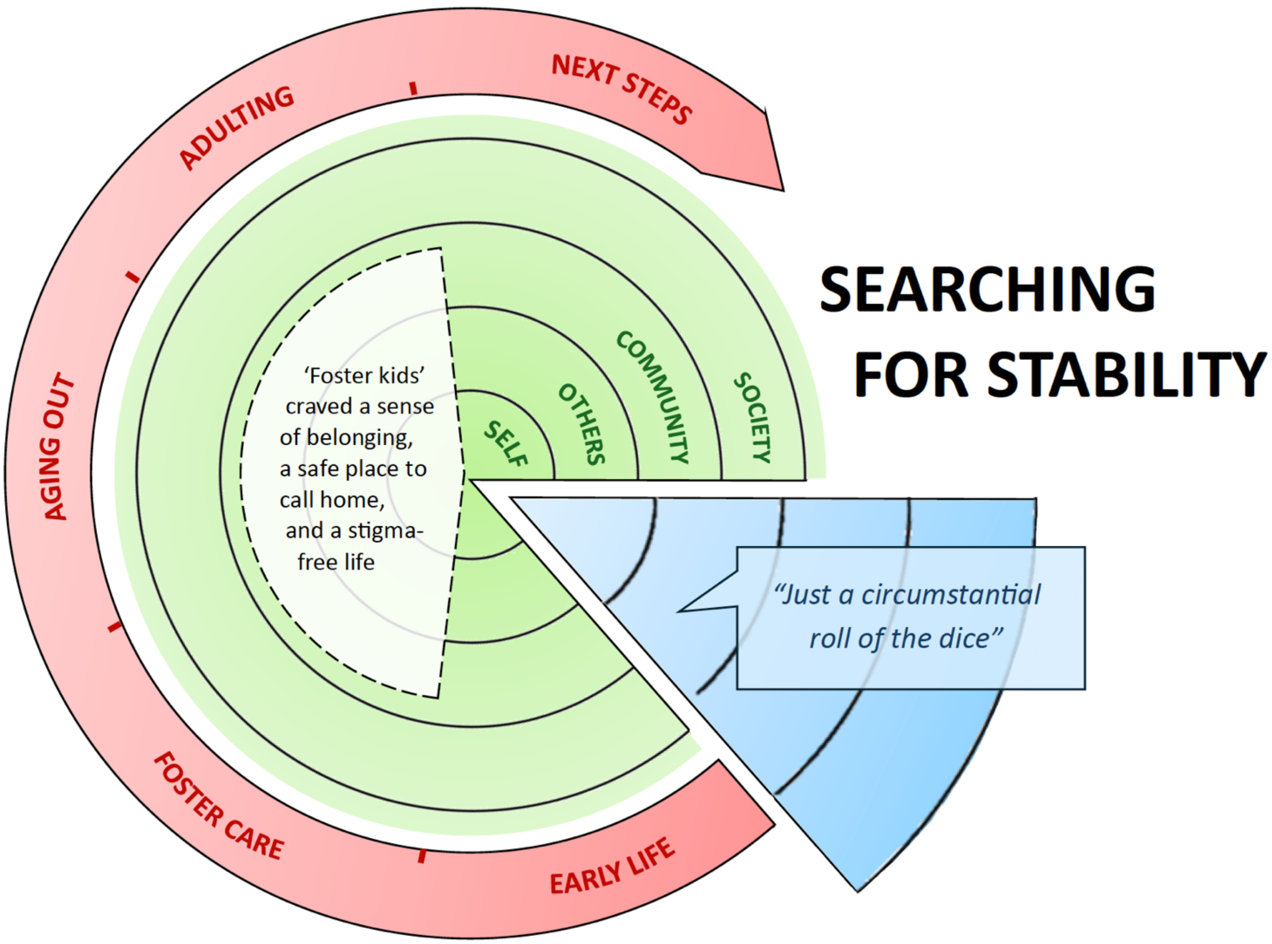

Supplemental Figure 3 Qualitative Theme: Helpers and Havens

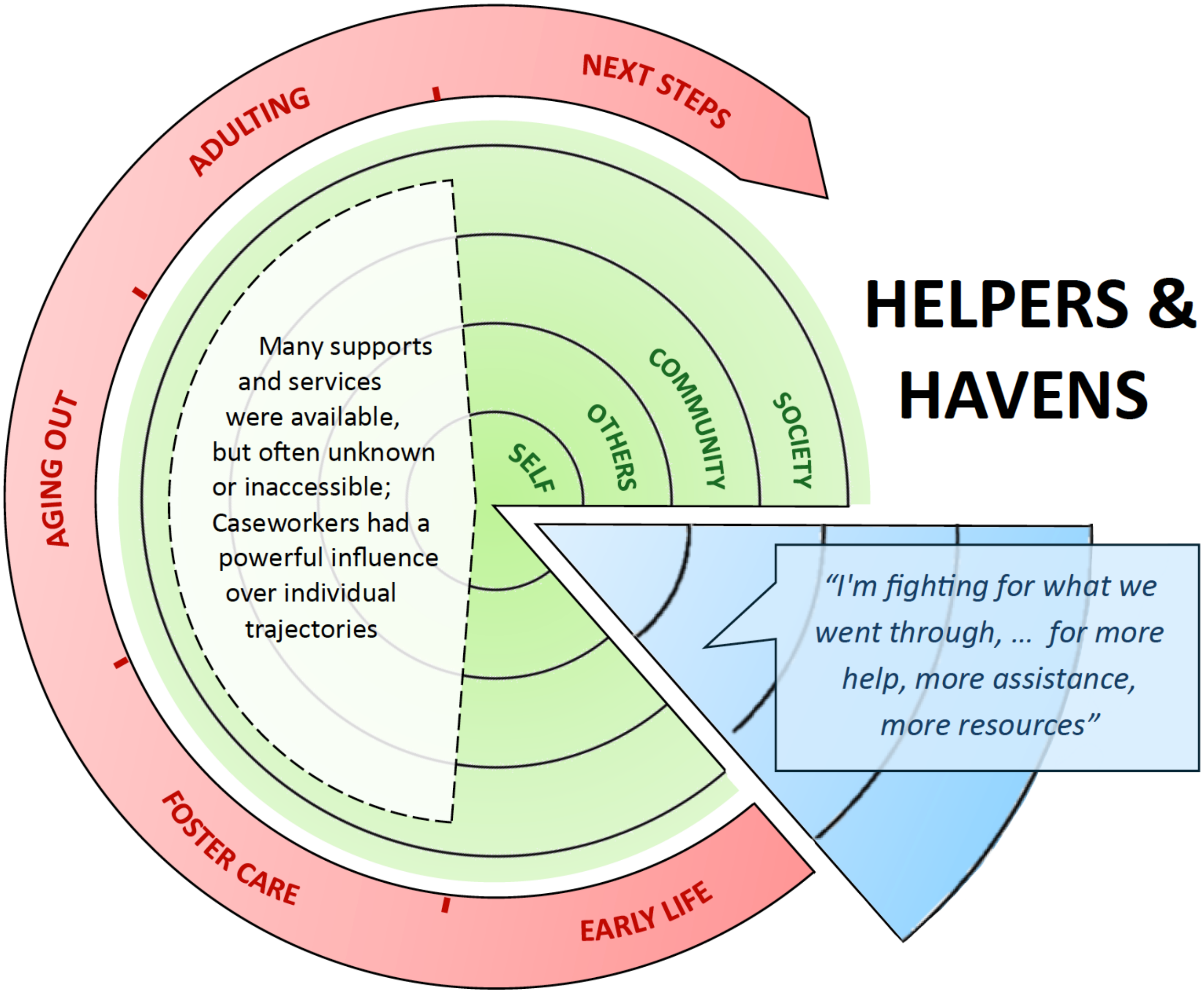

Supplemental Figure 4 Qualitative Theme: Aging Out and Adulting

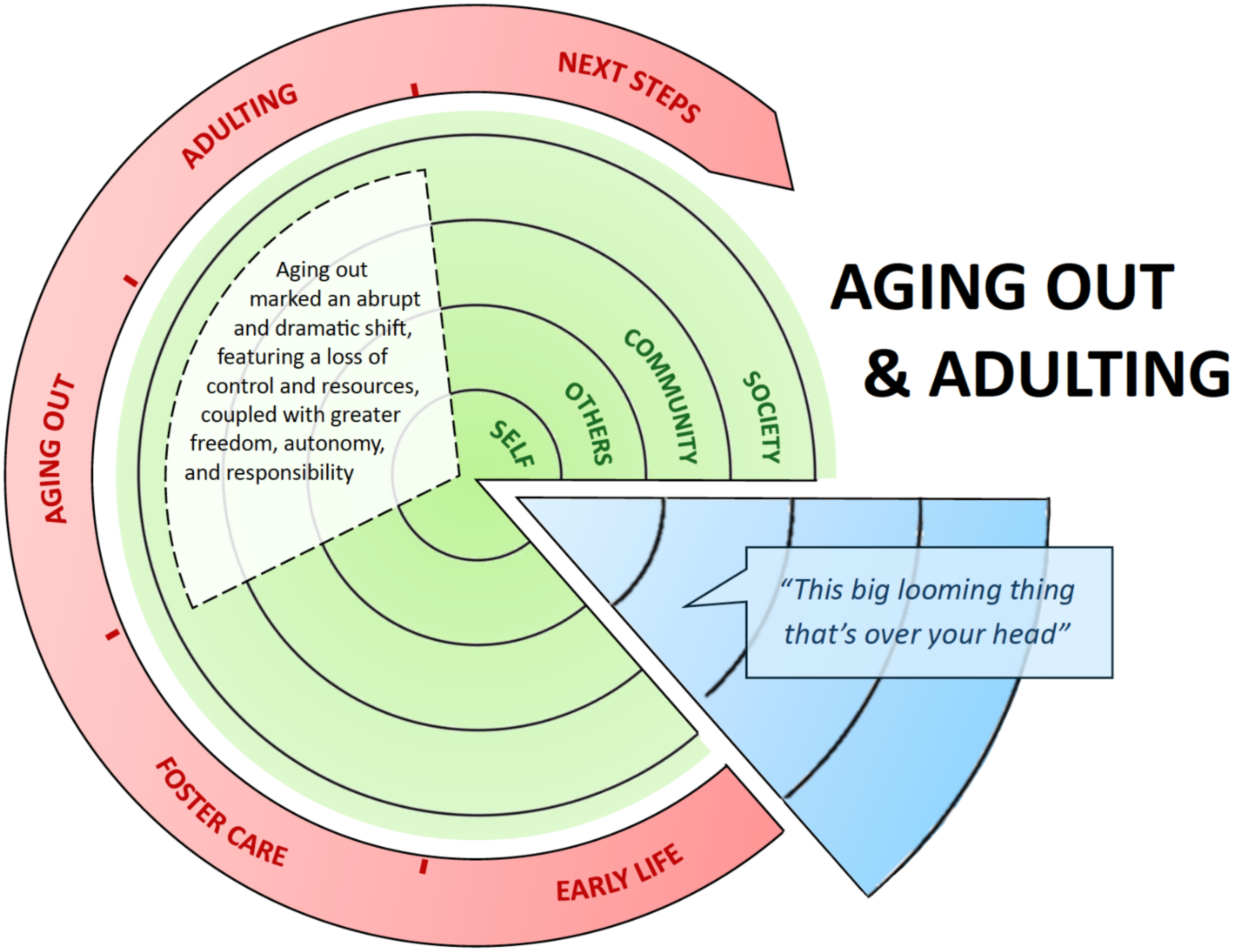

Supplemental Figure 5 Qualitative Theme: Relationships and Responsibilities

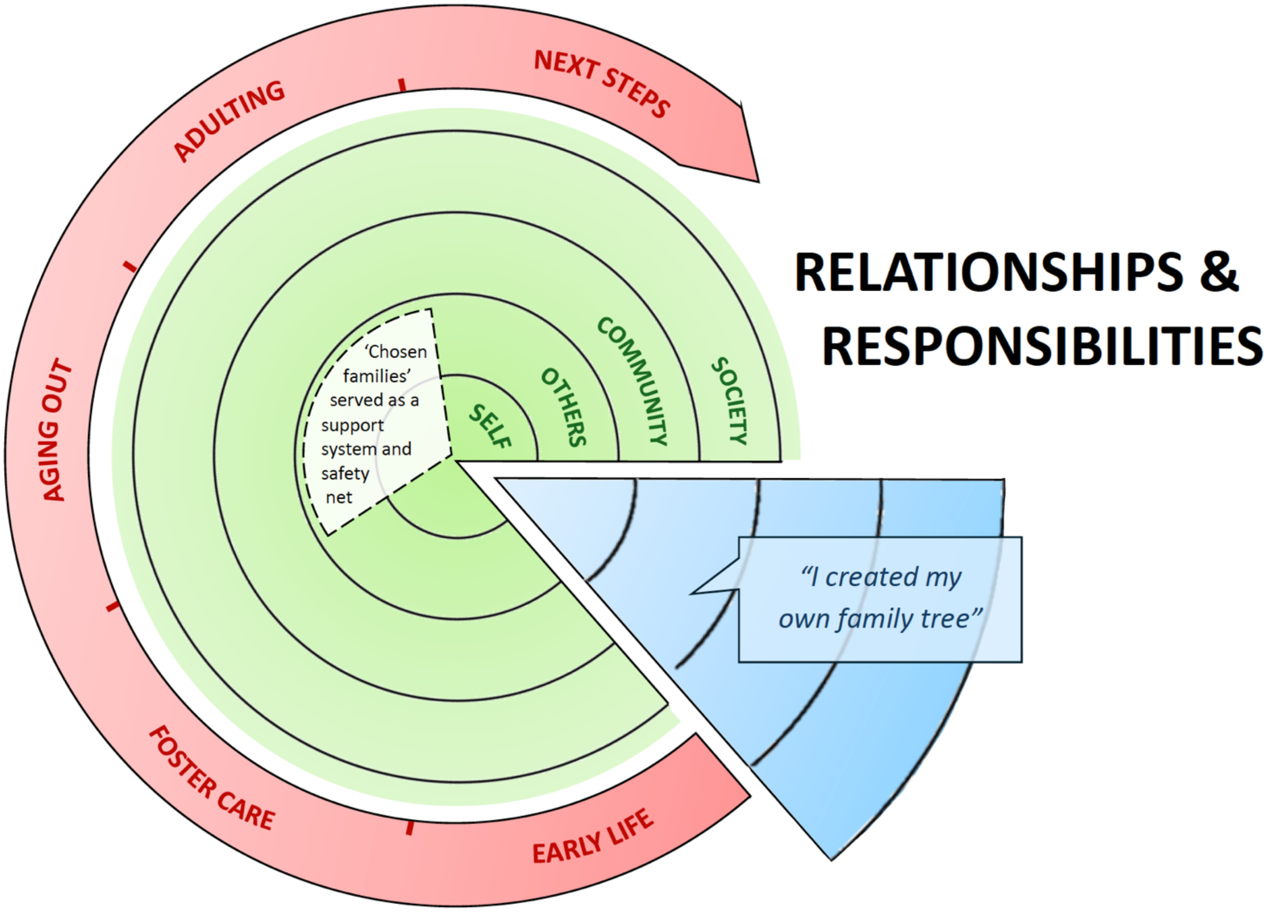

Supplemental Figure 6 Qualitative Theme: Health Challenges and Changes

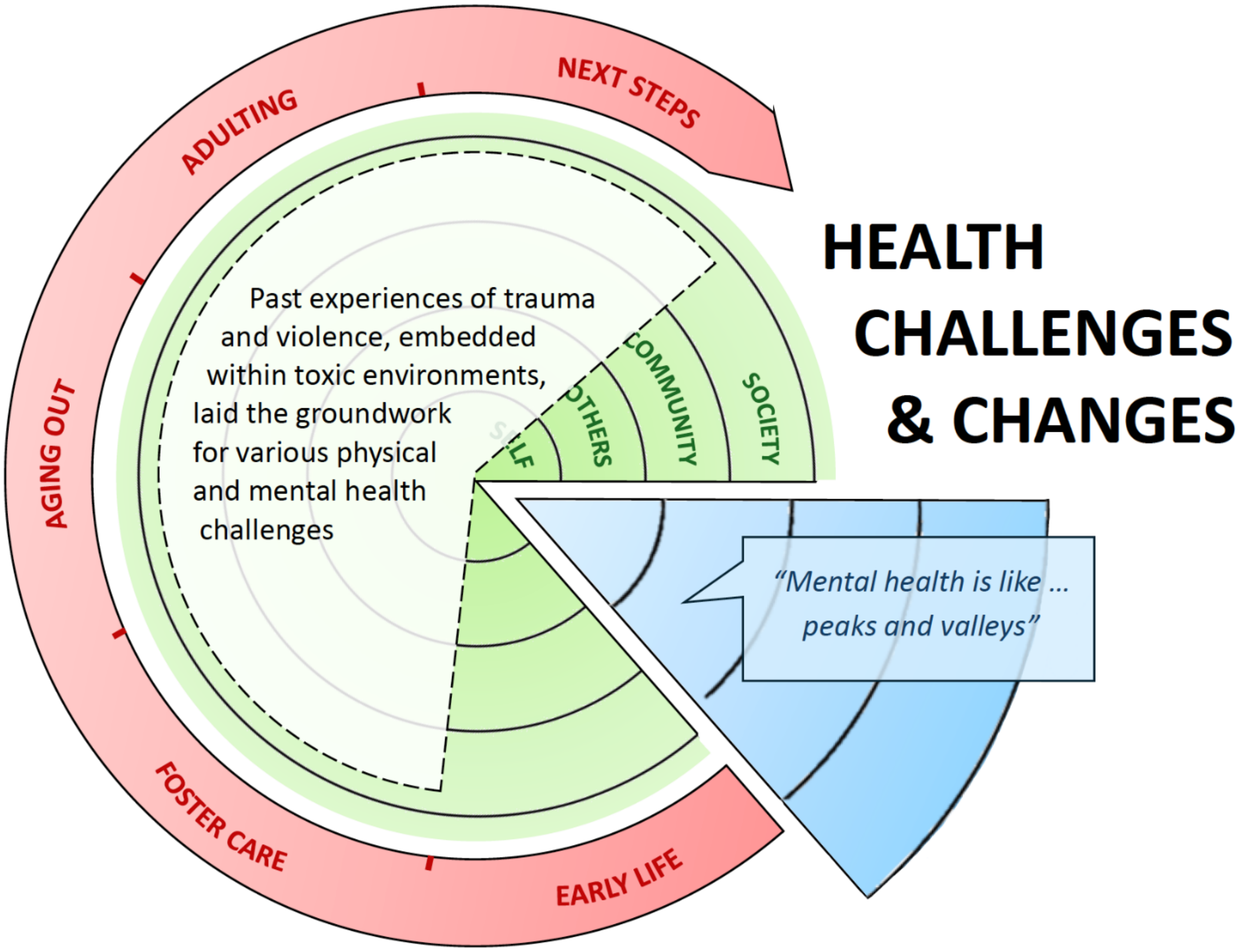

Supplemental Figure 7 Qualitative Theme: Remarkable Resilience

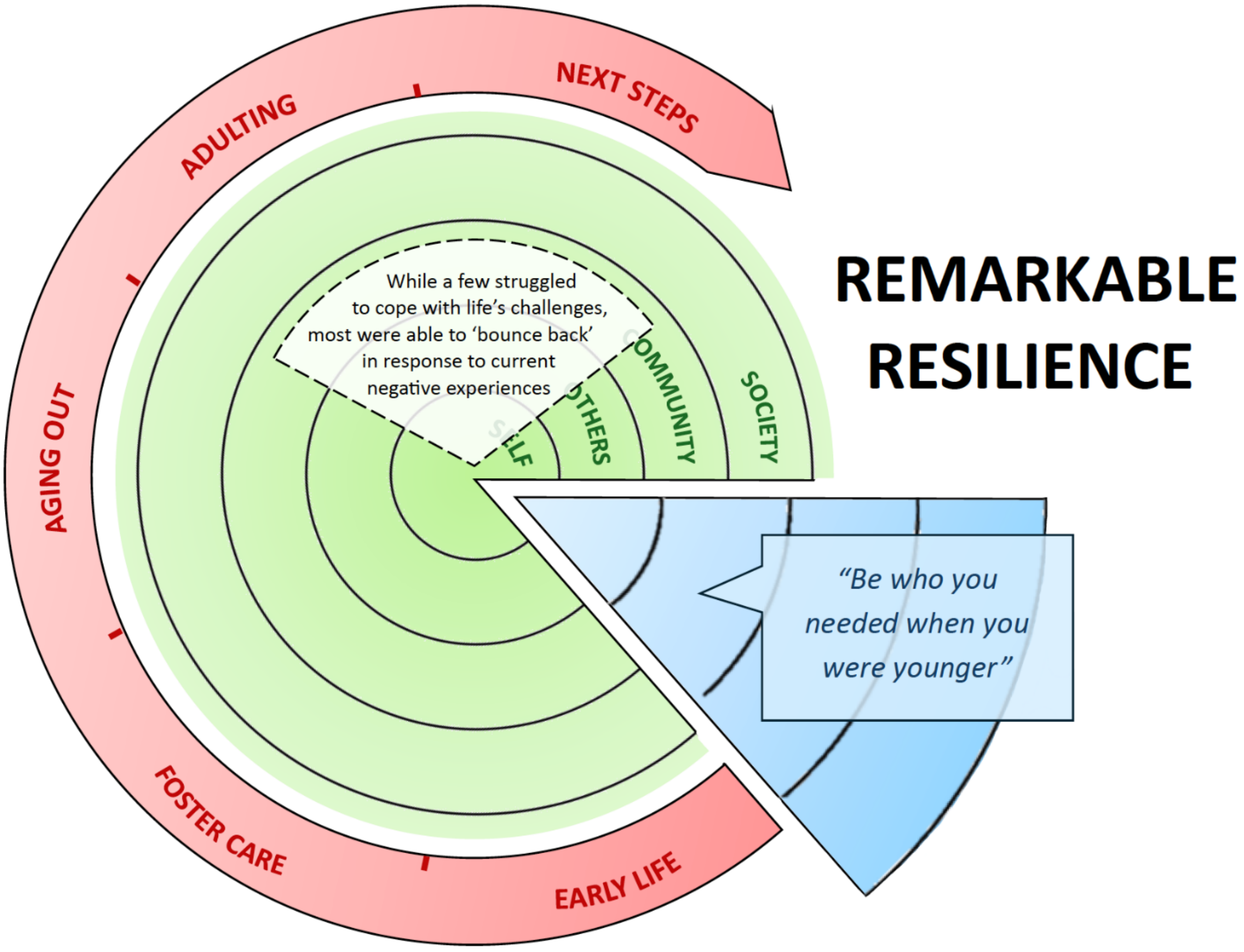

Supplemental Figure 8 Qualitative Theme: Moving Onward and Upward

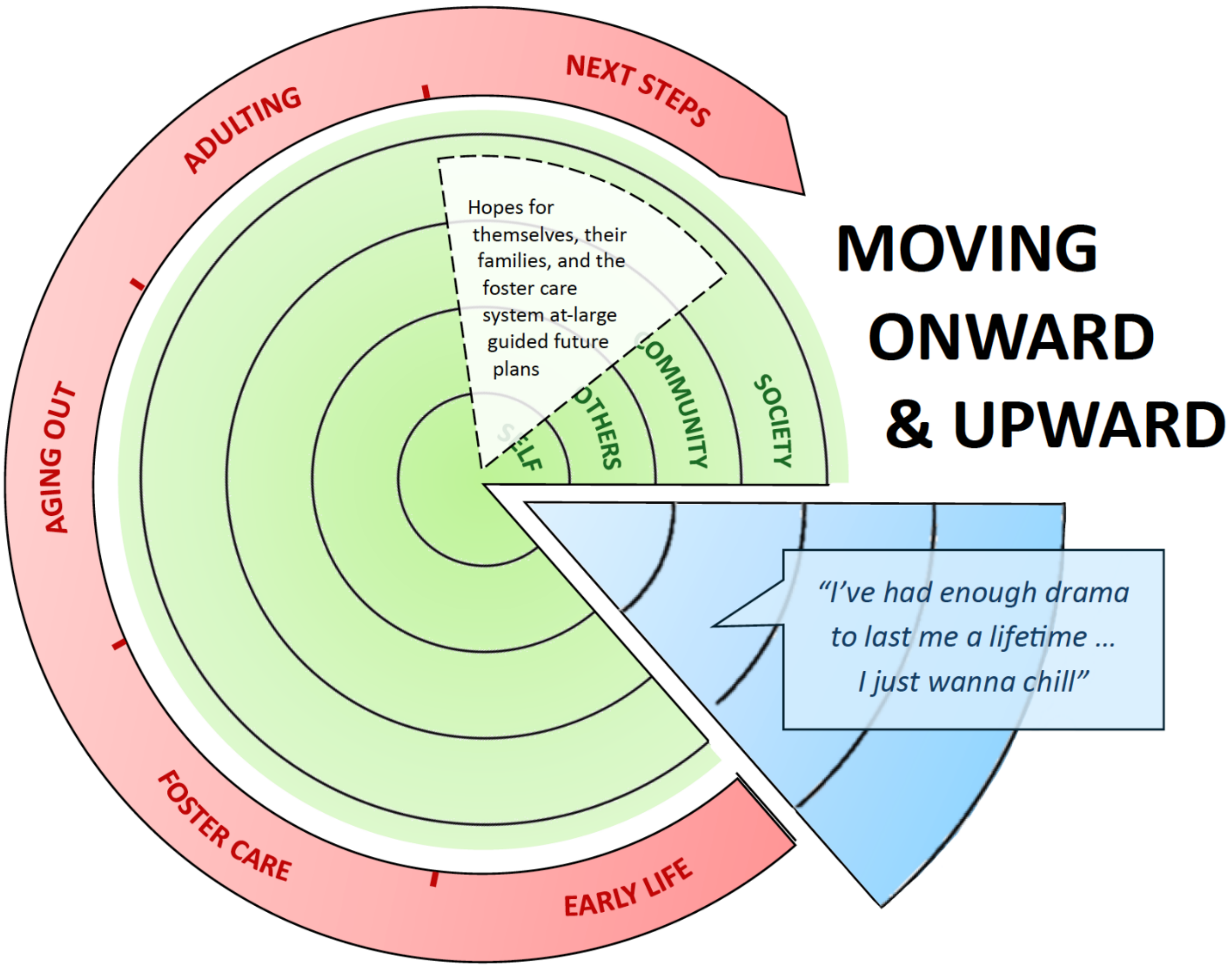

Supplement: S1 File — Block sequential regression tables and qualitative thematic diagrams. (PDF) [file pone.0338574.s001.pdf]
